# Investigation of 2‐stage meta‐analysis methods for joint longitudinal and time‐to‐event data through simulation and real data application

**DOI:** 10.1002/sim.7585

**Published:** 2017-12-18

**Authors:** Maria Sudell, Catrin Tudur Smith, François Gueyffier, Ruwanthi Kolamunnage‐Dona

**Affiliations:** ^1^ Department of Biostatistics University of Liverpool Liverpool UK; ^2^ Centre Hospitalier Universitaire de Lyon Lyon France

**Keywords:** joint model, longitudinal, meta‐analysis, simulation, time‐to‐event

## Abstract

**Background:**

Joint modelling of longitudinal and time‐to‐event data is often preferred over separate longitudinal or time‐to‐event analyses as it can account for study dropout, error in longitudinally measured covariates, and correlation between longitudinal and time‐to‐event outcomes. The joint modelling literature focuses mainly on the analysis of single studies with no methods currently available for the meta‐analysis of joint model estimates from multiple studies.

**Methods:**

We propose a 2‐stage method for meta‐analysis of joint model estimates. These methods are applied to the INDANA dataset to combine joint model estimates of systolic blood pressure with time to death, time to myocardial infarction, and time to stroke. Results are compared to meta‐analyses of separate longitudinal or time‐to‐event models. A simulation study is conducted to contrast separate versus joint analyses over a range of scenarios.

**Results:**

Using the real dataset, similar results were obtained by using the separate and joint analyses. However, the simulation study indicated a benefit of use of joint rather than separate methods in a meta‐analytic setting where association exists between the longitudinal and time‐to‐event outcomes.

**Conclusions:**

Where evidence of association between longitudinal and time‐to‐event outcomes exists, results from joint models over standalone analyses should be pooled in 2‐stage meta‐analyses.

## 1 INTRODUCTION

Univariate shared random effect joint models for longitudinal and time‐to‐event data simultaneously model a longitudinal and a time‐to‐event outcome.^1^ The model consists of a longitudinal submodel and a time‐to‐event submodel linked through an association structure, which quantifies the relationship between the 2 outcomes. A range of options exist in the literature for each submodel (including linear mixed effects models or splines for the longitudinal submodel and proportional hazards or accelerated failure time models for the time‐to‐event submodels). Various association structures have been used, including sharing just random effects between the submodels, sharing the current longitudinal trajectory (both the fixed and random effects), or sharing the first derivative or slope of the longitudinal trajectory. This investigation focuses on joint models that concern a single continuous longitudinal and a single possibly censored time‐to‐event outcome, linked using an association structure consisting of shared zero mean random effects with one common association parameter, termed proportional association.^2^

Methods for the joint analysis of longitudinal and time‐to‐event data are commonly used in analyses to account for study dropout and measurement error in time varying covariates, whilst producing less biased estimates of study parameters.^2^, ^3^ Powney et al 4 discuss the Magnetic trial,^5^ which reported a longitudinal case with missing data where a complete case analysis displayed no significant difference between treatment groups whereas use of joint models to account for missing data resulted in a statistically significant difference. Sudell et al 6 recently reported that the number of published joint analyses has been increasing over recent years, suggesting a growing resource of joint modelling investigations. Examples of joint models applied in the literature include Jacoby et al,^7^ Kolamunnage‐Dona et al 2016,^8^ Lloyd‐Williams et al 2014,^9^ and Kovanda et al 2017.^10^

Currently, the methodology available in the literature focuses on joint models in single study cases (for overviews see previous studies^3^, ^11^). If joint modelling methods present an improvement over separate longitudinal or time‐to‐event methods in a single study case under certain conditions (such as correlation between longitudinal and time‐to‐event outcomes, see Guo and Carlin 2004^12^), their use in a meta‐analytic setting may also be preferable over use of separate methods.

Meta‐analysis (MA) was defined by Glass^13^ as the statistical analysis or pooling of results from several studies. These methods can have increased precision and power compared to the original analyses, whilst allowing additional questions to be investigated. Meta‐analysis can be performed on the original data from each study, termed individual participant or patient data (IPD), or on study level results, termed aggregate data (AD). Methods for MA are described extensively in the literature (eg, Whitehead 2002^14^ and the Cochrane Handbook^15^). As a range of cases for single studies have been identified where joint models are preferably to separate longitudinal or time‐to‐event analyses, it follows that cases could exist where pooling joint model parameters is preferable to pooling separate model parameters.

In this article, we describe a method for pooling results from multiple joint longitudinal and time‐to‐event models fitted to data from different studies. The approach adopts a 2‐stage method, where IPD is modelled separately in each identified study and the model parameters pooled using standard MA techniques. Results of the proposed MA of joint model are compared to MAs of separate longitudinal or time‐to‐event analyses.

The article is organised as follows. We begin with a discussion of the methods used in this investigation. These methods are then applied to an example dataset. A simulation study is then presented, which compares the MA of joint and separate models under a range of situations. The report ends with a discussion of the use of joint models in a 2‐stage MA.

## 2 TWO STAGE META‐ANALYSIS OF JOINT‐MODEL DATA

The description of methods is split into those used in the first and the second stages of a 2‐stage MA. The first stage concerns fitting models to the IPD provided by each study in turn. The second stage then pools the estimates from these study‐specific model fits using standard meta‐analytic techniques.

### 2.1 Stage 1 – Modelling of data within studies

We fit joint longitudinal and time‐to‐event models to the data from each study. Usually, a joint model is formulated of 2 submodels; a longitudinal submodel and an event‐time submodel, and the 2 components are linked together through some association structure.

We considered a joint model where the longitudinal submodel consisted of a linear mixed effect model (Equation (1)).

(1) $$Y_{\mathrm{kij}}=X_{\mathrm{kij}}\beta_{1k}+Z_{\mathrm{kij}}b_{\mathrm{ki}}+\varepsilon_{\mathrm{kij}}$$

Throughout this article, *k* represents study membership, *i* a particular individual in study *k*, and *j* a particular time point at which their longitudinal outcome is measured. In Equation (1), the longitudinal outcome is represented by *Y*_*kij*_, and the design matrix for the fixed effects is represented by X_*kij*_ with corresponding population coefficients ***β***_1*k*_, which are estimated for each separate study *k*. The design matrix for the random effects is represented by *Z*_*kij*_, with corresponding individual specific random effects by ***b***_*ki*_. The random effects are assumed to follow a zero mean multivariate normal distribution *N*(0, Σ_k_). The error term is represented by *ε*_*kij*_ and is assumed to follow a $N\left(0,\sigma_{k}^{2}\right)$ distribution. The error terms and the random effects are considered independent of each other.

The time‐to‐event submodel of the joint model assumed the specification in Equation (2).

(2) $$\lambda_{\mathrm{ki}}\left(t\right)=\lambda_{0}\left(t\right)\exp\left(X_{\mathrm{ki}}\beta_{2k}+W_{2\mathrm{ki}}\left(t\right)\right)$$

In Equation (2), *λ*_0_(*t*) represents an unspecified baseline hazard and X_*ki*_ represents fixed time stationary population covariates with associated coefficients ***β***_2*k*_, which again are estimated for each separate study k. *W*_2*ki*_(*t*) represents the link between the time‐to‐event and the longitudinal submodels. We assumed a joint model that uses shared zero mean random effects with a proportional association. Specifically,

(3) $$W_{2\mathrm{ki}}\left(t\right)=\alpha_{k}W_{1\mathrm{ki}}\left(t\right)$$

Here *W*_1*ki*_(t) = *Z*_*ki*_***b***_*ki*_ are the zero mean random effects of the longitudinal submodel and *α*_*k*_ quantifies the association between the longitudinal and the time‐to‐event outcomes. Again, an association parameter *α* is estimated for each study *k* in the MA. Note that any instances of the longitudinal time variable in the association structure are replaced with the time‐to‐event time variable.

The separate longitudinal and time‐to‐event models have the same specifications as the longitudinal and time‐to‐event submodels of the joint model respectively, apart from that the separate time‐to‐event analysis model does not contain the *W*_2*ki*_(*t*) term.

### 2.2 Stage 2 – Pooling of results from studies

In this stage, parameters are extracted from the study‐specific models and pooled by using the standard MA methods. Both fixed and random MAs are performed for each analysis, and the results compared. The inverse variance method is used for both the fixed and random effects MA (with a DerSimonian and Laird^16^ approach for random MA, see Whitehead^14^ for a MA overview).

A fixed effects MA assumes that each study is estimating a common underlying effect denoted by *θ*. Any variability between studies is assumed to be attributable to sampling variability. The estimates from each study are then pooled by using a weighted average:

(4) $$\hat{\theta }=\frac{\sum_{k=1}^{K}w_{k}{\hat{\theta }}_{k}}{\sum_{k=1}^{K}w_{k}}$$

Here, *K* is the total number of studies, ${\hat{\theta }}_{k}$ is the effect estimate from the *k*th study for *k* = 1…*K*, $\hat{\theta }$ is the overall pooled effect estimate, and *w*_*k*_ is the weight for study *k*. For the inverse variance method, this is taken to be *w*_*k*_ = 1/*v*_*k*_ where $v_{k}=\mathrm{var}\left({\hat{\theta }}_{k}\right)$.

A random effects MA assumes that the observed effect estimates from each study are a random sample from the distribution of possible effect estimates, and pooled results estimate the mean effect and its variance for the population. If the variability between studies in effect estimates is represented by *τ*^2^, then the variance of the effect estimate is given by $\mathrm{var}\left({\hat{\theta }}_{k}\right)=v_{k}+\tau^{2}$. Results are then pooled again by using Equation (4), but with the weight term now equal to *w*_*k*_ = 1/(*v*_*k*_ + *τ*^2^).

### 2.3 Considerations

A variety of specifications exist for joint models in potential random effects and association structures.^17^ However, dependent on the model specified, association parameters can have very different interpretations.

For example, consider a joint model that uses an association structure that shares only zero mean random effects between the longitudinal and time‐to‐event submodels with a common association parameter (such that *W*_2*ki*_(*t*) = *α*_*k*_(*Z*_*ki*_***b***_*ki*_) **)**. Here, *α*_*k*_ quantifies how the deviation of individual *i* in study *k* from the population mean longitudinal trajectory at a given time affects their risk of an event.^17^ An example could be a case where individuals with higher blood pressure than the population average at a given time are at higher risk of a cardiac event.

Alternatively, a joint model that uses an association structure involving the true complete longitudinal trajectory with a common association parameter (such that *W*_2*ki*_(*t*) = *α*_*k*_(X_*ki*_***β***_1*k*_ + *Z*_*ki*_***b***_*ki*_)) has a different interpretation. Here, *α*_*k*_ quantifies how the value of the longitudinal outcome for individual *i* in study *k* at a given time point affects their risk of an event.^17^, ^18^ An example could be where the risk of a cardiac event for an individual increases as their recorded blood pressure increases.

The above 2 association parameters are modelling different types of links between the longitudinal and the time‐to‐event outcomes. Pooling the 2 association parameters described in the example above would assume that the risk of an event at a given time point because of the difference in the individual's recorded longitudinal value and the population mean and the risk of an event due to individual's recorded longitudinal value are the same.

Additionally, if the specification of the longitudinal submodel differs between joint models fitted, the terms linking the submodels in the association structure may differ, again leading to association parameters with differing interpretations. As an example, consider 2 joint models each linking the submodels using *W*_2*ki*_(*t*) = *α*_*k*_(*Z*_*ki*_***b***_*ki*_) with random effects specifications of random intercept alone *Z*_*ki*_***b***_*ki*_ = *b*_0*ki*_ or both random intercept and slope *Z*_*ki*_***b***_*ki*_ = *b*_0*ki*_ + *b*_1*ki*_time_*ki*_. In the first case, the *α*_*k*_ parameter quantifies the difference in risk of an event because of the difference between the population and individual specific intercept of the longitudinal trajectory. In the second, *α*_*k*_ quantifies the risk of an event due to differences between the individual and the population in both the intercept and the slope of the longitudinal trajectory.

As such, we note that joint models of differing specifications can produce association parameters with differing interpretations. Pooling association parameters with different interpretations assumes that these differences are unimportant, potentially a strong assumption. To avoid this assumption, we recommend pooling association parameters from joint models with identical association structures (family of association structure and the terms it involves). If differing joint models are appropriate between studies identified in a MA, we recommend subgrouping the studies, and fitting joint models of identical specification within but not across subgroups. This is reiterated in our recommendations for 2‐stage MA of joint data, see section 5.

In addition, a variety of options for the longitudinal and time‐to‐event submodels have been used in the literature.^6^ Pooling fixed effect coefficients from different types of submodel again assumes that their interpretation is comparable, which may not always be the case (such as for coefficients from proportional hazards models and accelerated failure time models). Again, care should be taken when using joint models in a 2‐stage analysis to only pool parameters with comparable interpretations.

### 2.4 Software and model fitting

During the simulation study, in the first stage, the joint models in this investigation were fitted by using the “joineR”^19^ package within the R programming language.^20^ Separate models were also fitted for comparison to the joint models; these were fitted by using the R package “nlme”,^21^ and the R package “survival”^22^ for the longitudinal and time‐to‐event data, respectively. For the real data application, joint models, and separate longitudinal and time‐to‐event models were fitted by using functions in the R package “joineRmeta” (available through GitHub: https://github.com/mesudell/joineRmeta).

When pooling study level results in the second stage, the R package “meta”^23^ was used. Separate MAs were conducted for each model parameter extracted. In this investigation, MAs were conducted for the longitudinal submodel treatment effect parameter *β*_12_, the time‐to‐event submodel treatment effect parameter *β*_21_, and the association parameter *α*. An example dataset and R code file to demonstrate the process of conducting a 2‐stage IPD‐MA of joint data are provided in the Supporting Information.

We should note that the joint models fitted from the “joineR” or the “joineRmeta” packages require bootstrapping procedures to estimate standard errors (Hsieh et al^24^ emphasised that use of the profile likelihood to evaluate standard errors in joint models could lead to underestimation of the standard errors when an unspecified baseline hazard is used). As such, the time taken to obtain the precision estimates from the joint model was considerably longer than for separate longitudinal or time‐to‐event analyses, an issue further examined in section 6.

## 3 APPLICATION

### 3.1 Example data

To assess the difference between separate longitudinal and time‐to‐event analyses and joint models in a real setting, we examined a subset of the INDANA dataset 25. The INDANA dataset consists of IPD from multiple studies and was assembled to determine whether the efficacy of pharmacological treatment for high blood pressure depends on the characteristics of the patient themselves. The subset we analysed consisted of data from 7 trials,^26^, ^27^, ^28^, ^29^, ^30^, ^31^, ^32^ which we henceforward refer to as the INDANA data. We refer to the individual studies in the subset as EWPHE,^26^ COOP,^27^ STOP,^28^ SHEP,^29^ SHPP,^30^ MRC1,^31^ and MRC2,^32^ respectively. INDANA contains hypertensive patients assigned to 1 of 2 treatment groups, namely, any treatment for hypertension, versus placebo, no treatment, or usual care. Longitudinal outcomes were systolic blood pressure (SBP) and diastolic blood pressure, respectively, with 3 possible time‐to‐event outcomes; time to death, time to myocardial infarction (MI), and time to stroke.

Possible measurement times for the longitudinal outcomes were at baseline, 6 months, 1 year, and then annually thereafter to a maximum of 7 years (giving 9 potential measurement times); however, measurement schedules differed between included studies. The SHPP trial provided no longitudinal information, the SHEP study recorded at least some individuals at 6 measurement times, and STOP and MRC1 recorded at least some individuals at 7 measurement times. The remaining studies reported measurements at all of the 9 possible measurement times. Only an individual's longitudinal measurements made before their recorded event time contributed towards each analysis. Tables of the number of measurements recorded for each study at each time point are available in Tables S1‐S3 in the Supporting Information.

We present a demonstration of methods applied to the longitudinally measured SBP and each time‐to‐event outcome. For EWPHE, an intention to treat analysis is only possible for fatal endpoints, and so we only utilised data from the study in the SBP and time to death analysis. Additionally, SHPP did not contribute to any analysis as it lacked any longitudinal information. Therefore, the final dataset contained a maximum of 6 studies totalling a maximum of 29 825 individuals. The exact number of individuals involved in each analysis depended on the amount of missing data for each outcome and are shown in Tables 1, 2, 3.

**Table 1:** Results for analysis of SBP and time to death from the INDANA data

Study	N (No. of Events) [No. Longitudinal Measurements]	Longitudinal: Treatment Coefficient *β*_12_ (95% CI)		Time‐to‐Event: Treatment Coefficient *β*_21_ (95% CI)		Association Parameter *α* (95% CI)
		Separate Analysis	Joint Model	Separate Analysis	Joint Model	Joint Model
Time to Death						
Estimates From Each Study						
COOP	884 (130) [4621]	−10.9 (−13.11, −8.69)	−10.9 (−13.3, −8.54)	−0.04 (−0.38, 0.31)	−0.05 (−0.46, 0.32)	−0.003 (−0.021, 0.012)
EWPHE	840 (284) [3825]	−11.38 (−13.44, −9.32)	−11.38 (−13.78, −9.09)	−0.1 (−0.33, 0.13)	−0.06 (−0.32, 0.18)	0.007 (−0.004, 0.018)
MRC1	17354 (501) [101078]	−8.18 (−8.57, −7.78)	−8.18 (−8.52, −7.81)	−0.03 (−0.2, 0.15)	0.02 (−0.18, 0.18)	0.034 (0.024, 0.042)
MRC2	4396 (616) [26457]	−10.66 (−11.31, −10.01)	−10.66 (−11.46, −10.07)	−0.04 (−0.19, 0.12)	−0.04 (−0.18, 0.11)	−0.001 (−0.013, 0.011)
SHEP	4736 (455) [20816]	−6.99 (−7.59, −6.38)	−6.99 (−7.64, −6.4)	−0.13 (−0.32, 0.05)	−0.11 (−0.31, 0.07)	0.008 (−0.001, 0.016)
STOP	1615 (96) [5777]	−11.94 (−13.27, −10.62)	−11.94 (−13.48, −10.43)	−0.51 (−0.92, −0.1)	−0.56 (−1.12, −0.15)	−0.011 (−0.04, 0.012)
Meta‐analysis						
Fixed MA	29825 (2082) [162574]	−8.66 (−8.94, −8.38)	−8.62 (−8.9, −8.34)	−0.08 (−0.17, 0)	−0.06 (−0.15, 0.03)	0.01 (0.01, 0.02)
Random MA	29825 (2082) [162574]	−9.86 (−11.4, −8.33)	−9.83 (−11.34, −8.32)	−0.09 (−0.17, 0)	−0.06 (−0.16, 0.03)	0.01 (−0.01, 0.02)
τ^2^		3.2091	3.0541	6.00E‐04	0.0015	2.00E‐04
I^2^		95.20%	94.90%	4.30%	10.90%	83.80%
*P* value		<.0001	<.0001	.3892	.3456	<.0001

*β*_21_, *β*_21_, and *α* are as defined in Equation (5); *P* value is for chi squared test for presence of significant heterogeneity.

**Table 2:** Results for analysis of SBP and time to MI from the INDANA data

Study	N (No. of Events) [No. Longitudinal Measurements]	Longitudinal: Treatment Coefficient *β*_12_ (95% CI)		Time‐to‐Event: Treatment Coefficient *β*_21_ (95% CI)		Association Parameter *α* (95% CI)
		Separate Analysis	Joint Model	Separate analysis	Joint Model	Joint Model
Time to MI						
Estimates From Each Study						
COOP	884 (73) [4573]	−11.1 (−13.32, −8.89)	−11.1 (−13.19, −8.7)	0.03 (−0.43, 0.49)	0.03 (−0.46, 0.4)	−0.001 (−0.019, 0.013)
MRC1	17354 (456) [100699]	−8.18 (−8.58, −7.79)	−8.18 (−8.61, −7.82)	−0.06 (−0.24, 0.12)	−0.02 (−0.19, 0.17)	0.032 (0.024, 0.041)
MRC2	4396 (287) [26303]	−10.7 (−11.36, −10.05)	−10.7 (−11.46, −10.14)	−0.21 (−0.44, 0.02)	−0.18 (−0.39, 0.04)	0.018 (0, 0.035)
SHEP	4728 (245) [20609]	−7.01 (−7.61, −6.4)	−7.01 (−7.63, −6.39)	−0.32 (−0.57, −0.06)	−0.31 (−0.62, −0.09)	0.003 (−0.011, 0.015)
STOP	1615 (63) [5739]	−11.96 (−13.28, −10.64)	−11.96 (−13.38, −10.52)	−0.23 (−0.72, 0.27)	−0.24 (−0.84, 0.26)	−0.004 (−0.035, 0.018)
Meta‐analysis						
Fixed MA	28977 (1124) [157923]	−8.62 (−8.91, −8.34)	−8.59 (−8.87, −8.3)	−0.16 (−0.27, −0.04)	−0.12 (−0.23, 0)	0.02 (0.01, 0.02)
Random MA	28977 (1124) [157923]	−9.66 (−11.32, −8.01)	−9.64 (−11.25, −8.02)	−0.16 (−0.27, −0.04)	−0.12 (−0.23, 0)	0.01 (0, 0.03)
*τ* ^2^		3.2083	3.0179	0	0	3.00E‐04
I^2^		96%	95.50%	0%	0.10%	83.20%
*P* value		<0.0001	<0.0001	0.4754	0.4053	<0.0001

*β*_21_, *β*_21_, and *α* are as defined in Equation (5); *P* value is for chi squared test for presence of significant heterogeneity.

**Table 3:** Results for analysis of SBP and time to stroke from the INDANA data

Study	N (No. of Events) [No. Longitudinal Measurements]	Longitudinal: Treatment Coefficient *β*_12_ (95% CI)		Time‐to‐Event: Treatment Coefficient *β*_21_ (95% CI)		Association Parameter *α* (95% CI)
		Separate Analysis	Joint Model	Separate Analysis	Joint Model	Joint Model
Time to Stroke						
Estimates From Each Study						
COOP	884 (59) [4527]	−10.73 (−12.94, −8.51)	−10.73 (−12.96, −8.33)	−0.58 (−1.12, −0.04)	−0.48 (−1.23, −0.02)	0.034 (0.016, 0.053)
MRC1	17354 (169) [100914]	−8.18 (−8.58, −7.79)	−8.18 (−8.59, −7.81)	−0.6 (−0.92, −0.29)	−0.51 (−0.87, −0.24)	0.061 (0.048, 0.072)
MRC2	4396 (235) [26251]	−10.66 (−11.31, −10)	−10.66 (−11.29, −9.98)	−0.28 (−0.54, −0.02)	−0.24 (−0.49, 0)	0.02 (−0.003, 0.039)
SHEP	4736 (262) [20441]	−6.96 (−7.56, −6.35)	−6.96 (−7.59, −6.45)	−0.45 (−0.7, −0.2)	−0.39 (−0.62, −0.15)	0.021 (0.01, 0.03)
STOP	1615 (83) [5701]	−11.7 (−13.03, −10.37)	−11.7 (−13.29, −10.15)	−0.64 (−1.1, −0.19)	−0.7 (−1.21, −0.24)	−0.014 (−0.038, 0.01)
Meta‐analysis						
Fixed MA	28985 (808) [157834]	−8.59 (−8.87, −8.3)	−8.56 (−8.84, −8.28)	−0.46 (−0.6, −0.32)	−0.39 (−0.53, −0.25)	0.03 (0.02, 0.04)
Random MA	28985 (808) [157834]	−9.53 (−11.14, −7.92)	−9.5 (−11.09, −7.91)	−0.46 (−0.6, −0.32)	−0.39 (−0.53, −0.25)	0.03 (0, 0.05)
τ^2^		3.0349	2.9163	0	0	6.00E‐04
I^2^		95.80%	95.70%	0%	0%	90.30%
*P* value		<.0001	<.0001	.4694	.4666	<.0001

*β*_21_, *β*_21_, and *α* are as defined in Equation (5); *P* value is for chi squared test for presence of significant heterogeneity.

During this investigation we aimed to demonstrate 2‐stage MA methods for joint data, not to investigate potential treatment modifiers in this dataset. As such, although the INDANA dataset contained additional patient covariates, models in this investigation only involved treatment assignment and longitudinal measurement time covariates.

It was noted that the trajectories of the longitudinal outcome SBP included a change point early on (see Figures S1‐S3 in the Supporting Information, which plot the individual and the mean longitudinal trajectories panelled by event type for each study). To account for this in the longitudinal submodel, a range of terms were tested, namely, $t_{\mathrm{ki}}^{2}$, exp(−*t*_*kij*_) and exp(−3^*^t_*kij*_). Through comparison of the log‐likelihoods and Akaike Information Criterion (AIC) values of the tested models, it was determined that inclusion of the term exp(−3^*^t_*kij*_) to account for the changepoint in the trajectory gave the best fit. As such, the joint models fitted to each study within the INDANA dataset had the following format:

(5) $$\begin{array}{l} Y_{\mathrm{kij}}=\beta_{10k}+\beta_{11k}t_{\mathrm{kij}}+\beta_{12k}{\text{treat}}_{\mathrm{ki}}+\beta_{13k}\exp\left(-3^{*}t_{\mathrm{kij}}\right)+b_{0i}+b_{1i}t_{\mathrm{kij}}+\varepsilon_{\mathrm{kij}}\\ \lambda_{\mathrm{ki}}\left(t\right)=\lambda_{0}\left(t\right)\exp\left(\beta_{21k}{\text{treat}}_{\mathrm{ki}}+W_{2\mathrm{ki}}\left(t\right)\right)\\ W_{2\mathrm{ki}}\left(t\right)=\alpha_{k}W_{1\mathrm{ki}}\left(t\right)=\alpha_{k}\left(b_{0i}+b_{1i}s_{\mathrm{ki}}\right) \end{array}$$

In the set of Equation 5, *t*_*kij*_ represents the measurement time for individual *i* in study *k* at the *j*th measurement time and treat_*ki*_ is their treatment assignment. Random effects are represented by *b* terms and the fixed effects by *β* terms. Fixed effects from the longitudinal and the time‐to‐event submodels are distinguished by the first element of the parameter's subscript, which takes a 1 or a 2 for the longitudinal or the time‐to‐event submodel, respectively. When linked to the time‐to‐event submodel, the *t*_*kij*_ covariate in the shared component is replaced with the survival time *s*_*ki*_.

The separate longitudinal and time‐to‐event models fitted for comparison to the joint model had the same format as the corresponding submodels of the joint model, with the *W*_2*ki*_(*t*) term not present in the separate time‐to‐event model.

In the models examined, a statistically significant negative treatment assignment coefficient in the time‐to‐event model (*β*_21_) would indicate that assignment to any treatment for hypertension versus placebo, no treatment, or usual care significantly reduced the risk of the event in question. A statistically significant negative treatment assignment coefficient in the longitudinal submodel (*β*_12_) would indicate that assignment to any treatment for hypertension significantly decreased SBP. A statistically significant positive association parameter (*α*) indicates that individuals with a positive deviation above the population mean longitudinal value in their recorded longitudinal values are at higher risk of the event at a given time point.

### 3.2 Results from INDANA

Tables 1, 2, 3 present results of the analysis of the INDANA dataset from the separate longitudinal and time‐to‐event analyses, and the joint model analyses, for the investigation of SBP and each of time to death, time to MI, and time to stroke. The results contain the study‐specific coefficient estimates, and their confidence intervals, for the longitudinal and time‐to‐event submodel treatment effects and the association parameters. The pooled results from both fixed and random MA are also included, along with heterogeneity statistics *τ*^2^, I^2^, and the *P* value for the chi squared test for presence of significant heterogeneity.^15^ Forest plots for the results are available in Figures S4‐S18.

The estimates for the longitudinal treatment effect coefficient were similar between the joint model and the separate longitudinal analysis (Tables 1, 2, 3). Specifically for each individual study, and for both the fixed and random MA pooled estimates, for each combination of SBP and time‐to‐event outcome examined, assignment to treatment for hypertension versus placebo, no treatment, or usual care was estimated to significantly reduce SBP. Some variability was identified between studies in the longitudinal outcomes (*τ*^2^ varying between 2.92 and 3.21, I^2^ statistic greater than 95% for all cases); however, both the fixed and random effects MAs agreed in the significance and direction of the results.

For the time‐to‐event treatment effect coefficient, we observed good agreement between the study‐specific estimates between the separate and joint analyses (Tables 1, 2, 3) for each set of outcomes. For time to death (Table 1), assignment to a hypertension treatment significantly decreased the risk of an event in the STOP trial. However, no significant effect of treatment was found in any other trial or in the pooled fixed or random MAs for either the separate or joint models. For time to MI (Table 2), a significant treatment effect was identified by both the separate and joint analyses in the SHEP trial, but no significant effect was identified by either method in the remaining trials. The pooled estimates for both the fixed and random MA for the separate time‐to‐event analysis showed a significant treatment effect whereby assignment to a treatment for hypertension reduced the risk of a MI. However, the pooled fixed or random MA estimate based on the joint model analysis was not significant. For time to stroke (Table 3), all studies for the separate analysis, and all but MRC2 for the joint analysis, reported a significant negative treatment effect. The pooled results for both methods were similar, with the fixed and random MA showing a significant pooled negative treatment effect. This indicated that both separate and joint methods showed that assignment to any hypertensive treatment versus no treatment, placebo, or usual care resulted in a decreased risk of stroke. There was little evidence of heterogeneity across any of the MAs for the time‐to‐event treatment effect coefficient, for any of time to death, MI, or stroke.

The association parameters estimated in each study for each set of analysis (SBP and time to death, SBP and time to MI, and SBP and time to stroke) indicate evidence of heterogeneity between the study estimates. For both time to death and time to MI (Tables 1, 2), a significant positive association parameter was estimated in the MRC1, the largest trial in the dataset. This resulted in a significant positive pooled association parameter estimate for the fixed MA for both time to death and time to MI; however, the pooled estimate from the random MA was not significant in either case. For time to stroke (Table 3), we estimated significant positive association parameters for studies COOP, MRC1, and SHEP. Again, the pooled estimate of the association parameter was significant and positive for the fixed MA but was not significant in the random MA. The larger confidence intervals reported in the random MA compared to the fixed MA was expected, given the high level of heterogeneity in the data. This difference in the estimated association parameters between studies may be attributable to the fact that this investigation did not include covariates in the model that are known to be linked to the outcomes of interest. For example, age is linked to blood pressure,^33^ and the mean age in MRC1 was 52.1 (SD 7.5), around 20 years lower than that of the other studies. A proportion of the observed heterogeneity in the association parameter may be explained by the covariates not currently included in the model. However, the focus of this investigation was to compare the behaviour of separate and joint modelling methods in MAs; investigations aimed to influence medical practice should assess additional covariates to those we included.

For this real data analysis, there was little difference in general between the estimates from the separate longitudinal or time‐to‐event analyses, and the joint model, apart from the difference in statistical significance of pooled estimates for the time‐to‐event treatment coefficient between the separate and joint analyses. The agreement between the separate and joint results is expected as the MAs of estimated association parameters showed evidence of heterogeneity, and the random effects MA gave pooled association parameters estimates not significantly different to 0. In single study cases, there is evidence that joint models provide less biased results in cases where the longitudinal and time‐to‐event outcomes are correlated.^12^ It is reasonable to assume that this behaviour persists in a multiple study case, although it may not have been apparent in our real data example.

## 4 SIMULATION STUDY

### 4.1 Methods

To further investigate the behaviour of MA of joint model, we conducted a simulation study. Data were simulated under the models shown in Equations (1)‐(3). In each scenario investigated, we simulated joint data consisting of a single continuous normal longitudinal outcome with a single time‐to‐event outcome for 5 studies, each containing 500 individuals randomised equally to 2 treatment groups. A maximum of 5 longitudinal measurements were recorded for each individual, at time points 0, 1, 2, 3, and 4, capped at their survival time.

The time‐to‐event outcome was simulated by using methods described in Bender^34^ and Austin.^35^ The event times for individuals were assumed to follow a Gompertz distribution with scale parameter equal to exp(*θ*_0_ + *αb*_0_) and shape parameter equal to *θ*_1_ + *αb*_1_. The extreme value distribution was used to calculate values for these parameters given that the mean value of the random effect terms is 0, and assuming a mean time of 3 and standard deviation of 1. We investigated data with high (~75%) and low (~25%) event rates. Event rates were controlled by using the exponentially distributed censoring times. After testing a range of parameter values, the *λ* parameter was set to exp(−3.08) for the high event rate and exp(−0.58) for the low event rate data.

Event times were generated in 2 stages to ensure censored event‐times. To begin, a random number *U*_*i*_ was generated from the uniform distribution *U*(0, 1) for each individual *i* in the simulated dataset. In the first stage, an indicator variable was assigned true if the both conditions below were satisfied and false otherwise:

$$\begin{array}{l} \text{Condition}\;1:\;\left(\theta_{1}+\alpha b_{1i}\right)<0\\ \text{Condition}\;2:\;U_{i}<\exp\left(\frac{\exp\left(\theta_{0}+\alpha b_{0i}\right)}{\theta_{1}+\alpha b_{1i}}\right) \end{array}$$

If the indicator variable was assigned true, the event time of that individual was set to infinite. Otherwise, a finite event time was calculated in the second stage by using Equation (7).

(6) $$T_{\mathrm{Ei}}=\frac{1}{\left(\theta_{1}+\alpha b_{1i}\right)}\log\left[1-\frac{\left(\theta_{1}+\alpha b_{1i}\right)\log\left(U_{i}\right)}{\exp\left(\theta_{0}+\alpha b_{0i}\right)\exp\left(\beta_{21}x_{21i}\right)}\right]$$

Here, *T*_*Ei*_ represents the *i*th individual's event time, *x*_21*i*_ represents their treatment assignment in the time‐to‐event submodel, and *β*_21_ the fixed effect coefficient for treatment assignment. The other parameters have been defined earlier.

The censoring times for each individual were then generated by using an exponential distribution^34^ by Equation 7.

(7) $$T_{\mathrm{Ci}}=-\frac{\log\left(U_{i}\right)}{\lambda }$$

Here, *T*_*Ci*_ represents the individual's censoring time. The final survival time for each individual was then calculated as *T*_*i*_ =  min (*T*_*Ei*_, *T*_*Ci*_), with the censoring variable recorded as 1 if *T*_*i*_ = *T*_*Ei*_ and 0 otherwise.

The variation in events rates between simulated studies was greater for high than low event rate data; for clarity, we report details of the event rates for different scenarios in Tables S4 and S5 in the Supporting Information.

Model parameters were chosen to represent relatively large differences between treatments, so the deviations of different methods from the true values could be clearly assessed. In the longitudinal submodel, the population intercept *β*_10_ was set to 1, *β*_11_ (the time coefficient) was set to 3, and *β*_12_ (the treatment assignment coefficient) was set to 2. The random effects followed a normal 0 mean multivariate distribution, with covariance matrix with on‐diagonals 0.9 (variation for the random intercept) and 1.2 (variation for the random slope), and off‐diagonals 0.5 (covariance between the random effects). The measurement errors ***ε***_*i*_ were assumed independent across time points and were generated from a 0 mean univariate normal distribution with variance 0.01. In the time‐to‐event submodel, the only covariate included was treatment assignment, which was assigned a coefficient of 3.

To fully investigate the behaviour of joint models compared to separate analyses, we investigated 5 association levels (*α* = 0, 0.25, 0.5, 0.75, and 1). We examined only positive associations as behaviour for negative associations is expected to be identical but in the opposite direction. Two sets of data were generated for each scenario, one with homogenous treatment effect and one with heterogeneous treatment effect across studies. For the homogenous treatment effect datasets, the longitudinal treatment effect coefficient was set to 2, whilst the time‐to‐event treatment effect coefficient was set to 3. For the heterogeneous treatment effect datasets, for each study, the longitudinal treatment effect coefficient was a realisation from *N*(2, 0.5), whilst the time‐to‐event treatment effect coefficient was a realisation from *N*(3, 0.5).

A total of 1000 simulations were run for each scenario. Within each simulation the modelling methods of interest (separate and joint analyses) were applied separately to each of the 5 simulated studies, and the results pooled in MAs using the methods discussed in section 2.2. From each simulation run, the pooled estimates of the longitudinal treatment effect coefficient, the time‐to‐event treatment effect coefficient, and the association parameter were extracted from both the fixed and random MAs. The *τ*^2^ parameter was also extracted, as well as the confidence intervals for each pooled estimate. During the simulation study, we recorded the number of failed fits (where the model failed for fit for at least one study in the simulation run) per scenario.

### 4.2 Simulation study results

**Figure 1:**
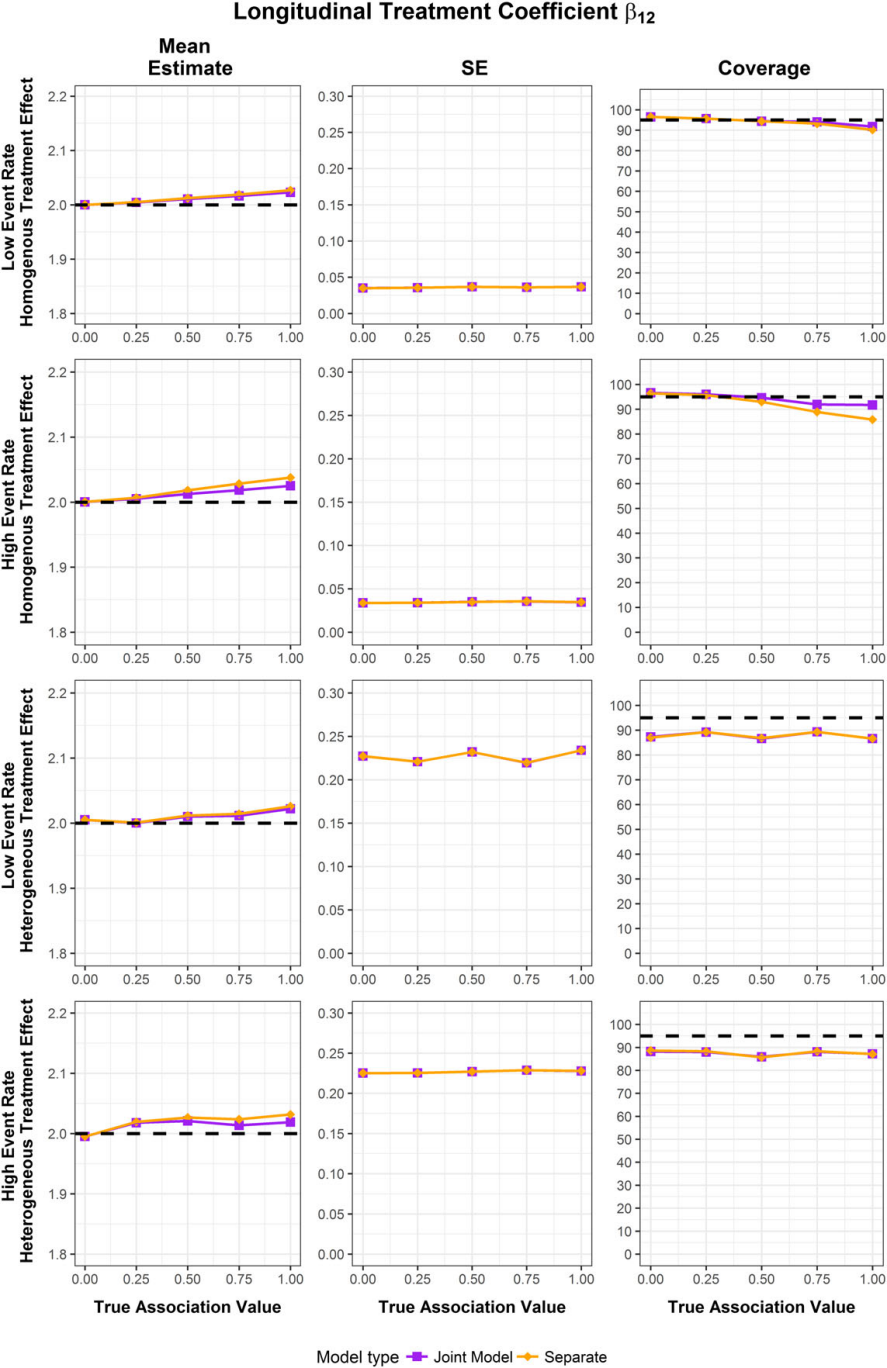
Mean estimate, standard error of estimates, and coverage for random effects MA of longitudinal treatment effect coefficient from 1000 simulation runs across different scenarios. The dotted line represents true value of coefficient for mean estimate, and the 95% coverage for the coverage groups [Colour figure can be viewed at http://wileyonlinelibrary.com]

**Figure 2:**
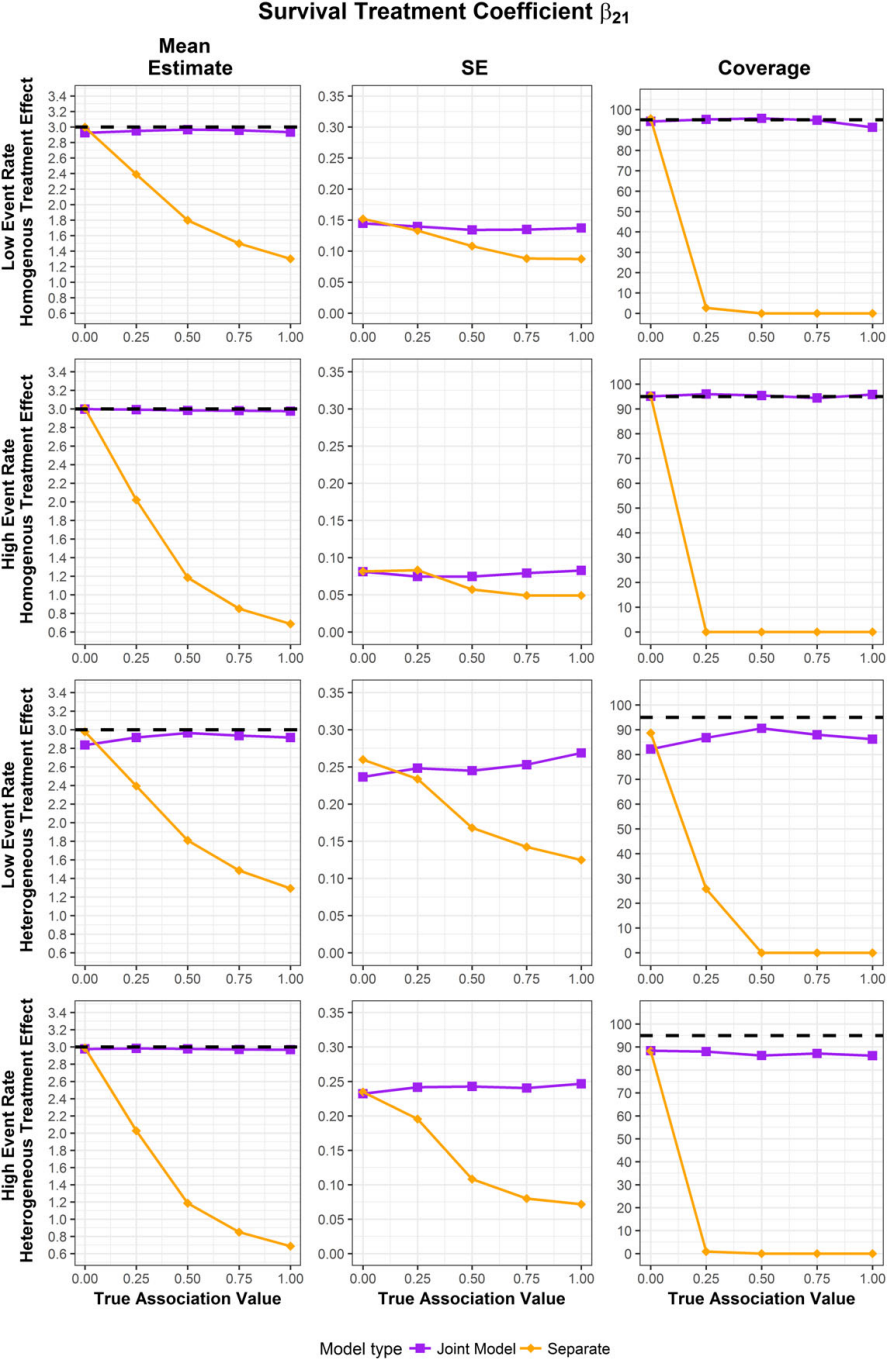
Mean estimate, standard error of estimates, and coverage for random MA of time‐to‐event treatment effect coefficient from 1000 simulation runs across different scenarios. The dotted line represents true value of coefficient for mean estimate, and the 95% coverage for the coverage groups [Colour figure can be viewed at http://wileyonlinelibrary.com]

**Figure 3:**
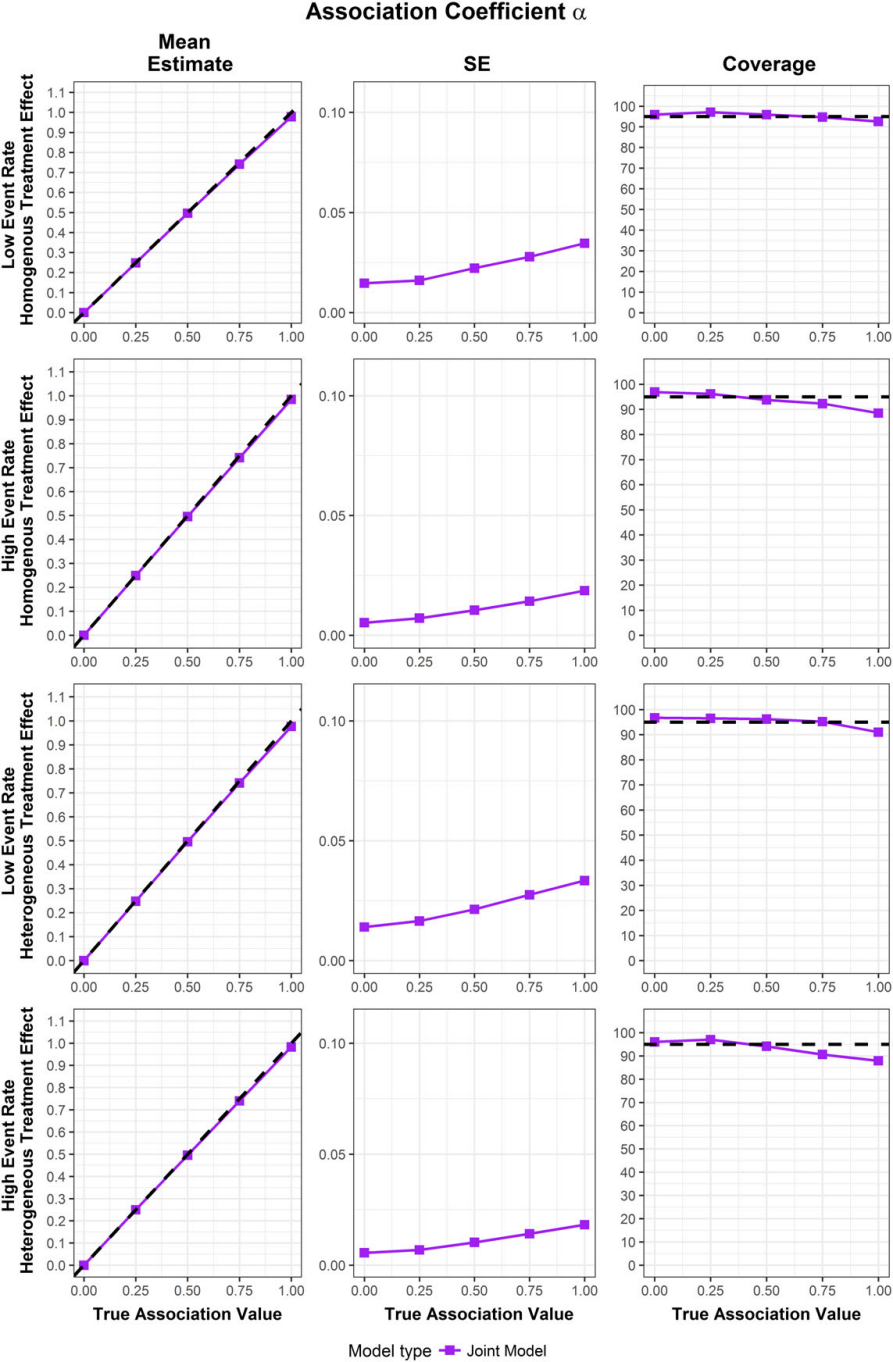
Mean estimate, standard error of estimates, and coverage for random MA of association parameter from 1000 simulation runs across different scenarios. The dotted line represents true value of coefficient for mean estimate, and the 95% coverage for the coverage group [Colour figure can be viewed at http://wileyonlinelibrary.com]

The results for the simulations are extensive, and so full results are presented in Tables S6‐S13 in the Supporting Information. Plots for the mean estimates, empirical standard error of the estimates (the standard deviation of the produced estimates), and coverages produced by the 1000 simulation runs for the longitudinal treatment coefficient, the time‐to‐event treatment coefficient, and the association parameter are shown in Figures 1, 2, 3, respectively. No failed fits were recorded.

The fixed and random effect MA results were similar for homogenous treatment effect scenarios, and the random effect MA showed better coverage for the heterogeneous treatment effect scenarios. As such, random MA simulation results are displayed in Figures 1, 2, 3, with fixed and random MA results available in Tables S6‐S13 in the Supporting Information.

The pooled mean estimates of the longitudinal treatment coefficient are similar from both the separate longitudinal model and the joint model across all simulation scenarios (Figure 1). Overall the treatment coefficient is estimated with little or no bias. The standard error and the coverages are also similar between the 2 methods, although lower for scenarios with heterogeneous treatment effect between studies.

In contrast, although the pooled mean estimates for the time‐to‐event treatment effect coefficient produced by the separate time‐to‐event analysis and joint model agree when the association between the longitudinal and time‐to‐event outcomes is not significant, once association is present, the separate and joint results appear markedly different (Figure 2). As strength of the association increases between the 2 outcomes, the separate time‐to‐event model underestimates the time‐to‐event treatment coefficient by an increasing amount, whilst the results from the joint model remain close to the true value. The empirical standard errors of the pooled estimates appears relatively constant from the joint models, with some increase as association increases. However, with increasing association, the empirical standard error of the pooled estimate from the separate time‐to‐event analysis decreases. There is a noticeable difference in the coverage values between the separate and joint approaches. The pooled estimates from the joint model show good coverage across scenarios. However, when an association between the longitudinal and time‐to‐event outcome is present, the coverage of the pooled mean estimate of the separate time‐to‐event analysis drops sharply.

Across all simulation scenarios, the pooled mean estimates for the association parameter were close to the true value from the joint model. The empirical standard error of the pooled estimates increases slightly with increasing association, and also the coverage decreases slightly for higher association levels. Future work should establish whether this behaviour persists under joint data simulated under different association structures, for example, in a joint model where the link between submodels depends on both fixed and random effects.

## 5 RECOMMENDATIONS FOR TWO STAGE MA OF INDIVIDUAL PARTICIPANT OR PATIENT DATA

In this section, we outline recommendations for researchers to consider when structuring a 2‐stage MA of joint longitudinal and time‐to‐event IPD.

### 5.1 Preliminary work

- For each study in the MA plot the longitudinal trajectories separately for those experiencing an event and those censored.
- Using these plots, identify whether there is an association between the longitudinal outcome and event‐time (for example, whether individuals experiencing events have higher recorded values of the longitudinal outcomes compared to censored individuals).
- Determine the type of association structure that best suits the aims of your analysis. For example, if clinically, the amount of deviation between an individual and the population average in the longitudinal trajectory is thought to effect the risk of an event, then a zero mean random effects only sharing structure should be used.^2^ Alternatively, if clinical evidence exists suggesting that the rate of change of the longitudinal trajectory affects the risk of an event, then the first derivative of the longitudinal trajectory should be shared between submodels.^18^ A wide range of association structures exist, the choice of which should have a clinical motivation. Overviews of association structures are available in the literature.^17^

### 5.2 First stage

- Group the studies identified in the MA so that the chosen model structure within each group (type of longitudinal submodel, type of time‐to‐event submodel, association structure, random effect specification, and if both fixed and random effects are shared between submodels specification of fixed effects in the longitudinal submodel) is identical. This is to ensure comparable interpretations of model parameters.
- Within each group, fit identical joint models to data from each study. Model structures can differ between groups.

### 5.3 Second stage

- For each study *k* extract parameters (eg, treatment assignment coefficients from each submodel (*β*_12*k*_, *β*_21*k*_) and association parameter (*α*_*k*_)) from the model fit, along with estimates of their precisions, and the sample size of the study. Additional parameter information can be extracted if they are of interest.
- Pool estimates within each group using the inverse variance methods described in section 2.2. Perform MA separately for each longitudinal coefficient, time‐to‐event coefficient and association parameter of interest.
- Results between groups with different joint model specifications can be qualitatively compared in discussion of the MA

## 6 DISCUSSION

This investigation highlighted several areas to be considered when joint models are used in 2‐stage MAs, when IPD is available to the investigator. We note, however, that the methods described for the second stage of this process could equally be applied during a MA of study level or AD, given that sufficient information was available to ensure that only parameters with comparable interpretations were pooled.

In the real data example, we noted little difference between the separate longitudinal or time‐to‐event analyses, and the joint analyses. This could be attributable to the pooled association from the random MA parameters being insignificant (examined in preference to the fixed MA due to evidence of heterogeneity; Table 1, 2, 3 and Figures S8, S13, and S18 in the Supporting Information). However, as shown by the simulation study, significant stronger association with higher risk could lead to more apparent differences between pooled results from separate time‐to‐event analyses and joint analyses.

We noted that fixed and random MA methods generally gave similar results for the homogenous simulated datasets, but that results differed more for the heterogeneous treatment simulated datasets. This suggests that researchers conducting a MA by using joint longitudinal and time‐to‐event data should, as with other MA, ensure that random effects methods are used if it is known or suspected that heterogeneity exists between the trials included in the MA. This can be achieved through examination of forest plots, and exploring estimates of the *τ*^2^ parameter and I^2^ statistic.

Throughout the simulation study, the coverage for the joint model methods appeared consistently high across different scenarios and association levels. Examination of the estimates from the model fits in the simulation study highlighted that the joint methods consistently give pooled estimates close to the true values across scenarios for association parameters or treatment effects from either submodel. Further, the simulation study also identified that separate methods for the longitudinal component appear adequate across scenarios investigated, for any association level examined.

However, whilst separate time‐to‐event methods give little bias for pooled treatment effect where there is insignificant association between the time‐to‐event and longitudinal outcomes, where an association is present the treatment effect is underestimated, with bias increasing as the strength of association increases. This behaviour might be linked to the case where omission of covariates from Cox models leads to bias in estimated effect parameters.^36^, ^37^, ^38^ Compared to the joint time‐to‐event submodel (Equation (2)), in the separate time‐to‐event models the *W*_2*ki*_(*t*) term is not included. Where association is present, ie, when *α*_*k*_ ≠ 0, the joint analysis models risk of an event associated with the longitudinal outcome via *W*_2*ki*_(*t*). This term (which has an effect on the event risk) is not included in the separate time‐to‐event model, potentially explaining the observed biased treatment effect estimates. This behaviour was not observed between the separate and joint longitudinal analyses as the model specifications for the longitudinal trajectory are identical in both models.

We have noted earlier the overall time taken to fit and extract information from joint model fits being larger than for separate analyses because of the bootstrapping requirement of drawing standard errors in the “joineR” and “joineRmeta” software. This issue is even more apparent for 2‐stage MAs as separate model fittings are required for multiple studies. During our application to real data, model fitting and bootstrapping of 200 samples have taken over 12 hours in a standard computing environment. To improve the situation, we have used the University of Liverpool's HTCondor system (see Litzkow et al,^39^ https://research.cs.wisc.edu/htcondor/, and http://condor.liv.ac.uk/ which was also used to run the simulations). Researchers without access to such specialised computing systems would have to experience long running times on standalone computers to fit joint models relying on bootstrapping for estimating the standard errors.

Guo and Carlin^12^ analysed a single dataset by using separate and joint models. The association parameter in the joint model was significant and negative. The differences observed between the estimated survival times produced by the 2 approaches were attributed to the fact that the joint models accounted for correlation between the longitudinal and time‐to‐event outcomes, whereas the separate time‐to‐event model did not. In our investigation, we observed a similar result for the multi‐study case—where an association between the longitudinal and time‐to‐event outcomes existed, the pooled estimates from the separate time‐to‐event model underestimated the true simulated time‐to‐event treatment effect compared to the pooled estimates from the joint model. However, where the association was insignificant, the separate and joint analyses produced similar results. In addition, the coverage for the treatment effect coefficient was noticeably better from the joint model estimates than the separate analysis where an association existed. The pooled results from the longitudinal separate model gave similar results to the pooled results from the joint methods regardless of the level of association between longitudinal and time‐to‐event outcomes. Therefore, as with single study cases, in a 2‐stage meta‐analytic case, there is evidence of benefit of joint methods over separate methods for estimation of time‐to‐event coefficients where an association between the longitudinal and time‐to‐event outcomes is known or suspected.

We should again highlight the difference in association structures in joint models available to researchers, which leads to an important concept to consider in both IPD 2‐stage MA, and in aggregate data meta‐analyses (AD‐MA), where study level results are obtained from published information or other sources. During a MA involving joint models, we recommend that the association structure should be kept consistent across included studies, to be able to pool the association estimates. If the most appropriate joint modelling structure (random effect specification, association structure, and longitudinal fixed effects if both fixed and random effects are shared between submodels) differs between identified studies, studies should be grouped by joint model structure, and only results from identical joint models (within groups) should be pooled. Pooled estimates from different groups (different joint modelling structures) can then by qualitatively compared in the discussion of the MA, whilst bearing in mind their potentially different interpretation. We have clarified out recommendations for the 2‐stage MA of joint longitudinal and time‐to‐event data in section 5.

There remain a wide range of areas to investigate in the 2‐stage MA of joint data. This investigation held sample size and association strength constant across studies within each MA in the simulation study. In our real data example, we observed varying sample sizes and association parameters between studies (the latter potentially due to differences in study demographics). Further work investigating a wider range of scenarios on the 2‐stage MA of joint data would be beneficial. In addition, further work examining and comparing the association structures available to researchers would be informative.

In the future, we recommend that researchers use joint models in place of separate longitudinal or time‐to‐event models to analyse joint longitudinal and time‐to‐event data in a meta‐analytic setting, if a potential association is suspected between longitudinal and time‐to‐event outcomes, as evaluated by producing plots of the longitudinal trajectories, stratified by event type (see section 5). Whilst adopting a joint modelling approach is more computationally intensive, we have demonstrated that a separate time‐to‐event approach underestimated the true time‐to‐event treatment effect when an association between the outcomes existed, with reduced coverage for the estimated coefficient. However, as separate and joint methods gave similar MA results in simulations and real data analyses when association is insignificant, to minimise resources used separate methods could be justified. The choice of model (association structure, baseline hazard, random effect specification, time‐to‐event and longitudinal submodel, etc.) should be made based on the requirements of each individual investigation, as one association structure could prove to be more appropriate than others.

## DATA ACCESSIBILITY

## Supporting information

Figure S1: Longitudinal trajectory plots with mean trajectory smoother (red line) for systolic blood pressure (SBP) and time to death data, with 0 indicating a censoring and 1 indicating an event was experienced.
Figure S2: Longitudinal trajectory plots with mean trajectory smoother (red line) for systolic blood pressure (SBP) and time to myocardial infarction (MI) data, with 0 indicating a censoring and 1 indicating an event was experienced.
Figure S3: Longitudinal trajectory plots with mean trajectory smoother (red line) for systolic blood pressure (SBP) and time to stroke data, with 0 indicating a censoring and 1 indicating an event was experienced.
Figure S4: Forest plot for longitudinal treatment effect covariate from standalone/separate longitudinal model for systolic blood pressure (SBP) and time to death
Figure S5: Forest plot for longitudinal treatment effect covariate from joint model for systolic blood pressure (SBP) and time to death
Figure S6: Forest plot for time‐to‐event treatment effect covariate from standalone/separate time‐to‐event model for systolic blood pressure (SBP) and time to death
Figure S7: Forest plot for time‐to‐event treatment effect covariate from joint model for systolic blood pressure (SBP) and time to death
Figure S8: Forest plot for association parameter from joint model for systolic blood pressure (SBP) and time to death
Figure S9: Forest plot for longitudinal treatment effect covariate from standalone/separate longitudinal model for systolic blood pressure (SBP) and time to myocardial infarction (MI)
Figure S10: Forest plot for longitudinal treatment effect covariate from joint model for systolic blood pressure (SBP) and time to myocardial infarction (MI)
Figure S11: Forest plot for time‐to‐event treatment effect covariate from standalone/separate time‐to‐event model for systolic blood pressure (SBP) and time to myocardial infarction (MI)
Figure S12: Forest plot for time‐to‐event treatment effect covariate from joint model for systolic blood pressure (SBP) and time to myocardial infarction (MI)
Figure S13: Forest plot for association parameter from joint model for systolic blood pressure (SBP) and time to myocardial infarction (MI)
Figure S14: Forest plot for longitudinal treatment effect covariate from standalone/separate longitudinal model for systolic blood pressure (SBP) and time to stroke
Figure S15: Forest plot for longitudinal treatment effect covariate from joint model for systolic blood pressure (SBP) and time to stroke
Figure S16: Forest plot for time‐to‐event treatment effect covariate from standalone/separate time‐to‐event model for systolic blood pressure (SBP) and time to stroke
Figure S17: Forest plot for time‐to‐event treatment effect covariate from joint model for systolic blood pressure (SBP) and time to stroke
Figure S18: Forest plot for association parameter from joint model for systolic blood pressure (SBP) and time to stroke
Table S1: Number of longitudinal measurements available at each time point by study for the analysis of systolic blood pressure (SBP) and time to death
Table S2: Number of longitudinal measurements available at each time point by study for the analysis of systolic blood pressure (SBP) and time to myocardial infarction (MI)
Table S3: Number of longitudinal measurements available at each time point by study for the analysis of systolic blood pressure (SBP) and time to stroke
Table S4: Mean and median event rates for homogeneous scenarios
Table S5: Mean and median event rates for heterogeneous treatment scenarios
Table S6: Two stage simulation results homogenous low event rate data joint model (joineR)
Table S7: Two stage simulation results homogenous low event rate data separate models
Table S8: Two stage simulation results homogenous high event rate data joint model (joineR)
Table S9: Two stage simulation results homogenous high event rate data separate models
Table S10: Two stage simulation results heterogeneous treatment low event rate data joint model (joineR)
Table S11: Two stage simulation results heterogeneous treatment low event rate data separate models
Table S12: Two stage simulation results heterogeneous treatment high event rate data joint model (joineR)
Table S13: Two stage simulation results heterogeneous treatment high event rate data separate models
