# Supplementary material for: Investigation of 2‐stage meta‐analysis methods for joint longitudinal and time‐to‐event data through simulation and real data application
Source: Stat Med. 2017 Dec 18;37(8):1227–44. doi: 10.1002/sim.7585 (PMC5887954; doi:10.1002/sim.7585)
Supplement: Supplementary file 1 — Figure S1: Longitudinal trajectory plots with mean trajectory smoother (red line) for systolic blood pressure (SBP) and time to death data, with 0 indicating a censoring and 1 indicating an event was experienced. Figure S2: Longitudinal trajectory plots with mean trajectory smoother (red line) for systolic blood pressure (SBP) and time to myocardial infarction (MI) data, with 0 indicating a censoring and 1 indicating an event was experienced. Figure S3: Longitudinal trajectory plots with mean trajectory smoother (red line) for systolic blood pressure (SBP) and time to stroke data, with 0 indicating a censoring and 1 indicating an event was experienced. Figure S4: Forest plot for longitudinal treatment effect covariate from standalone/separate longitudinal model for systolic blood pressure (SBP) and time to death Figure S5: Forest plot for longitudinal treatment effect covariate from joint model for systolic blood pressure (SBP) and time to death Figure S6: Forest plot for time‐to‐event treatment effect covariate from standalone/separate time‐to‐event model for systolic blood pressure (SBP) and time to death Figure S7: Forest plot for time‐to‐event treatment effect covariate from joint model for systolic blood pressure (SBP) and time to death Figure S8: Forest plot for association parameter from joint model for systolic blood pressure (SBP) and time to death Figure S9: Forest plot for longitudinal treatment effect covariate from standalone/separate longitudinal model for systolic blood pressure (SBP) and time to myocardial infarction (MI) Figure S10: Forest plot for longitudinal treatment effect covariate from joint model for systolic blood pressure (SBP) and time to myocardial infarction (MI) Figure S11: Forest plot for time‐to‐event treatment effect covariate from standalone/separate time‐to‐event model for systolic blood pressure (SBP) and time to myocardial infarction (MI) Figure S12: Forest plot for time‐to‐event treatment effect covariate from joint model for s [file SIM-37-1227-s001.zip › Supplementary Information.docx]

Supplementary Information

# Plots of Trajectories panelled by event type

## SBP and time to death


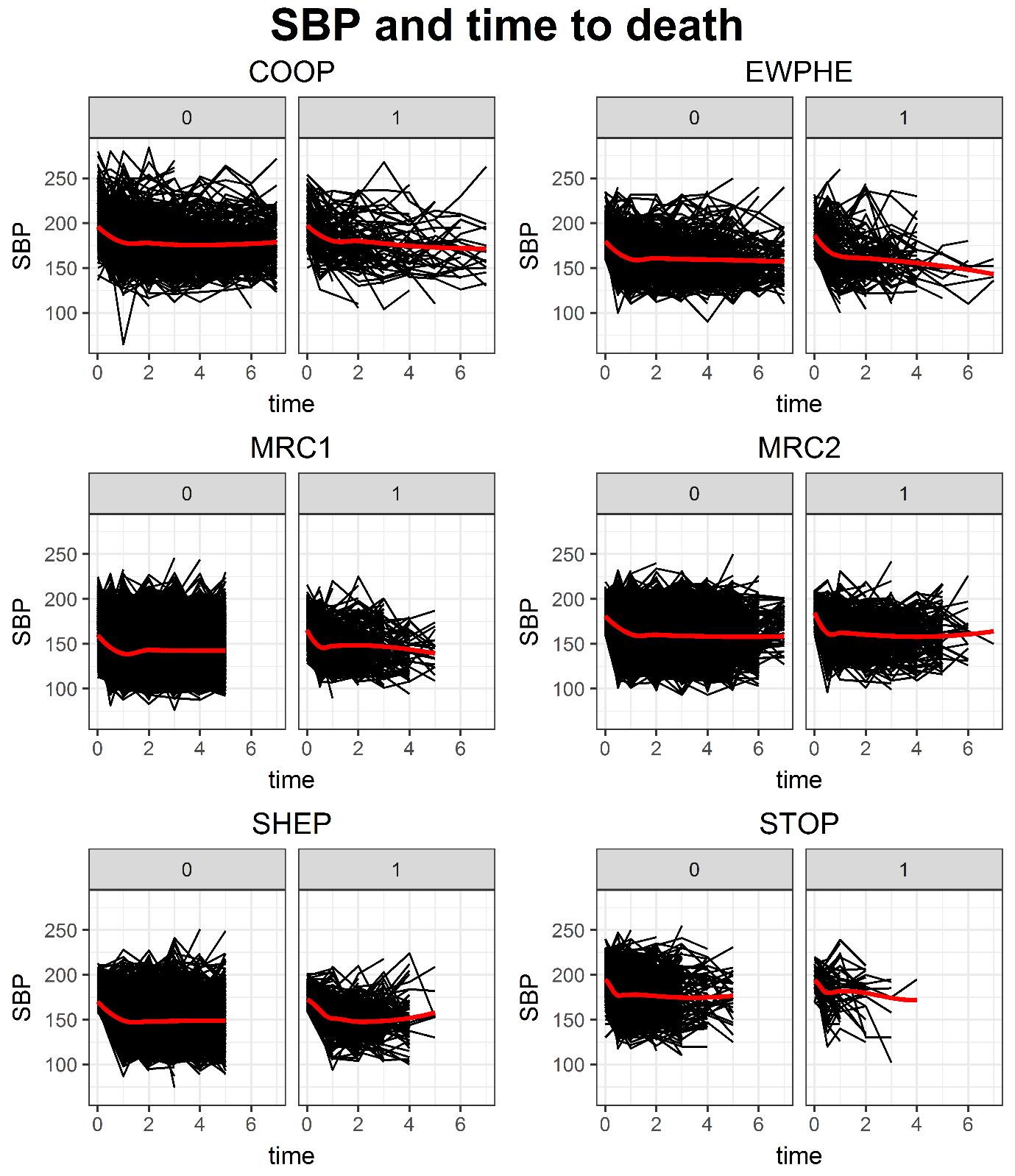


Supplemental Figure 1: Longitudinal trajectory plots with mean trajectory smoother (red line) for SBP and time to death data, with 0 indicating a censoring and 1 indicating an event was experienced.

## SBP and time to MI


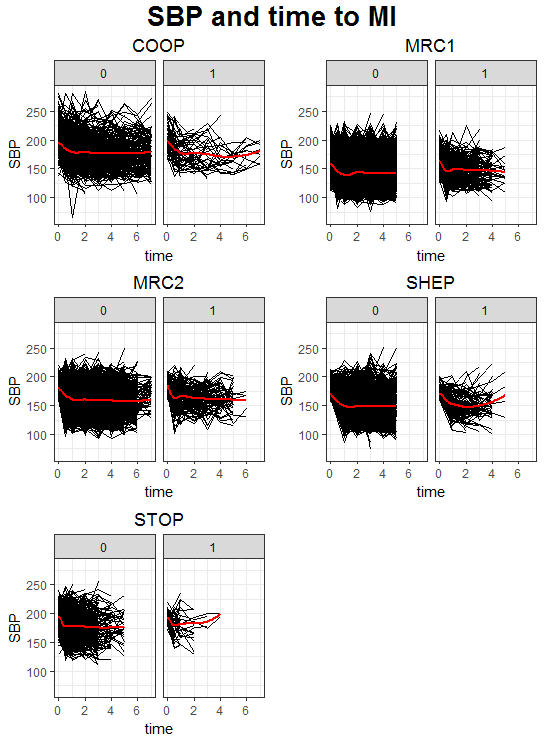


Supplemental Figure 2: Longitudinal trajectory plots with mean trajectory smoother (red line) for SBP and time to myocardial infarction (MI) data, with 0 indicating a censoring and 1 indicating an event was experienced.

## SBP and time to stroke


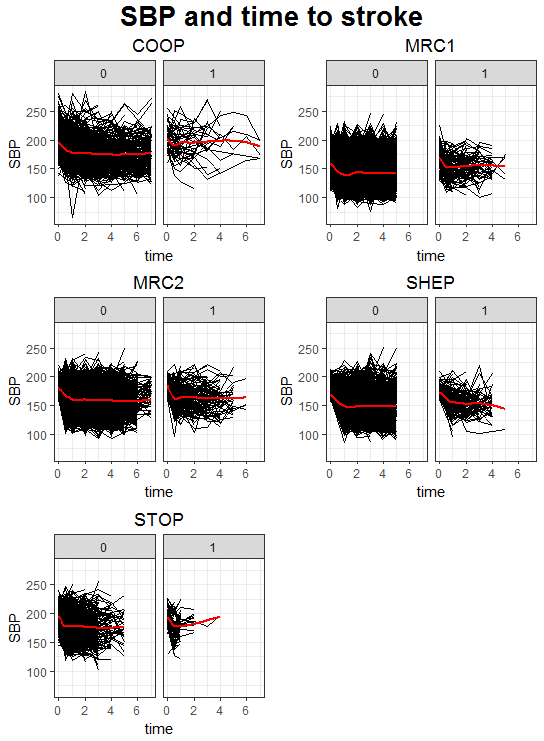


Supplemental Figure 3: Longitudinal trajectory plots with mean trajectory smoother (red line) for SBP and time to stroke data, with 0 indicating a censoring and 1 indicating an event was experienced.

# Table of number of longitudinal measurements at each time point

| SBP and time to death | | Time (years) | | | | | | | | | Number of measurement times recorded in study |
| --- | --- | --- | --- | --- | --- | --- | --- | --- | --- | --- | --- |
|  |  | 0 | 0.5 | 1 | 2 | 3 | 4 | 5 | 6 | 7 |  |
| Study | COOP | 884 | 760 | 785 | 722 | 514 | 329 | 267 | 200 | 160 | 9 |
|  | EWPHE | 840 | 749 | 653 | 509 | 383 | 297 | 213 | 118 | 63 | 9 |
|  | MRC1 | 17354 | 16525 | 16343 | 15308 | 14611 | 12584 | 8353 | 0 | 0 | 7 |
|  | MRC2 | 4394 | 4182 | 4100 | 3765 | 3490 | 3223 | 2596 | 655 | 52 | 9 |
|  | SHEP | 4736 | 0 | 4243 | 4091 | 3938 | 2644 | 1164 | 0 | 0 | 6 |
|  | STOP | 1612 | 1520 | 1440 | 798 | 311 | 67 | 29 | 0 | 0 | 7 |

Supplemental Table 1: Number of longitudinal measurements available at each time point by study for the analysis of SBP and time to death

| SBP and time to MI | | Time (years) | | | | | | | | | Number of measurement times recorded in study |
| --- | --- | --- | --- | --- | --- | --- | --- | --- | --- | --- | --- |
|  |  | 0 | 0.5 | 1 | 2 | 3 | 4 | 5 | 6 | 7 |  |
| Study | COOP | 884 | 759 | 782 | 713 | 506 | 319 | 260 | 195 | 155 | 9 |
|  | MRC1 | 17354 | 16512 | 16309 | 15253 | 14520 | 12478 | 8273 | 0 | 0 | 7 |
|  | MRC2 | 4394 | 4176 | 4086 | 3739 | 3465 | 3183 | 2564 | 644 | 52 | 9 |
|  | SHEP | 4728 | 0 | 4220 | 4051 | 3877 | 2592 | 1141 | 0 | 0 | 6 |
|  | STOP | 1612 | 1518 | 1433 | 784 | 299 | 65 | 28 | 0 | 0 | 7 |

Supplemental Table 2: Number of longitudinal measurements available at each time point by study for the analysis of SBP and time to myocardial infarction (MI)

| SBP and time to stroke | | Time (years) | | | | | | | | | Number of measurement times recorded in study |
| --- | --- | --- | --- | --- | --- | --- | --- | --- | --- | --- | --- |
|  |  | 0 | 0.5 | 1 | 2 | 3 | 4 | 5 | 6 | 7 |  |
| Study | COOP | 884 | 754 | 777 | 709 | 496 | 316 | 251 | 187 | 153 | 9 |
|  | MRC1 | 17354 | 16521 | 16325 | 15282 | 14572 | 12542 | 8318 | 0 | 0 | 7 |
|  | MRC2 | 4394 | 4172 | 4080 | 3737 | 3451 | 3177 | 2547 | 642 | 51 | 9 |
|  | SHEP | 4736 | 0 | 4206 | 4005 | 3834 | 2555 | 1105 | 0 | 0 | 6 |
|  | STOP | 1612 | 1515 | 1418 | 767 | 294 | 66 | 29 | 0 | 0 | 7 |

Supplemental Table 3: Number of longitudinal measurements available at each time point by study for the analysis of SBP and time to stroke

# Forrest plots for real data analyses

## SBP and time to death

### Separate longitudinal treatment effect


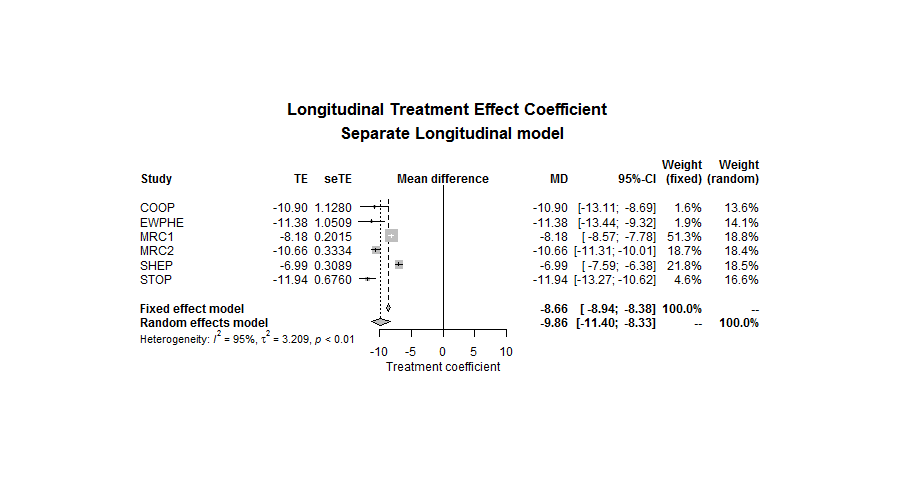


Supplemental Figure 4: Forest plot for longitudinal treatment effect covariate from standalone / separate longitudinal model for SBP and time to death

### Joint longitudinal treatment effect


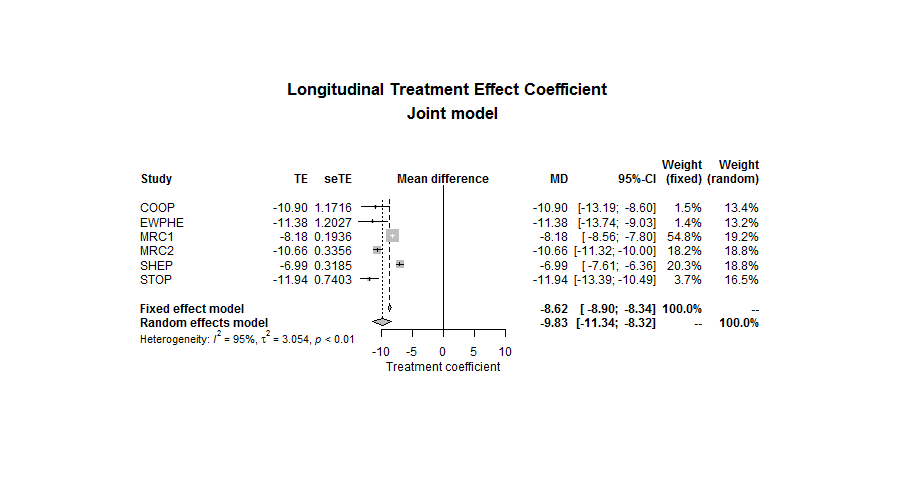


Supplemental Figure 5: Forest plot for longitudinal treatment effect covariate from joint model for SBP and time to death

### Separate time-to-event treatment effect


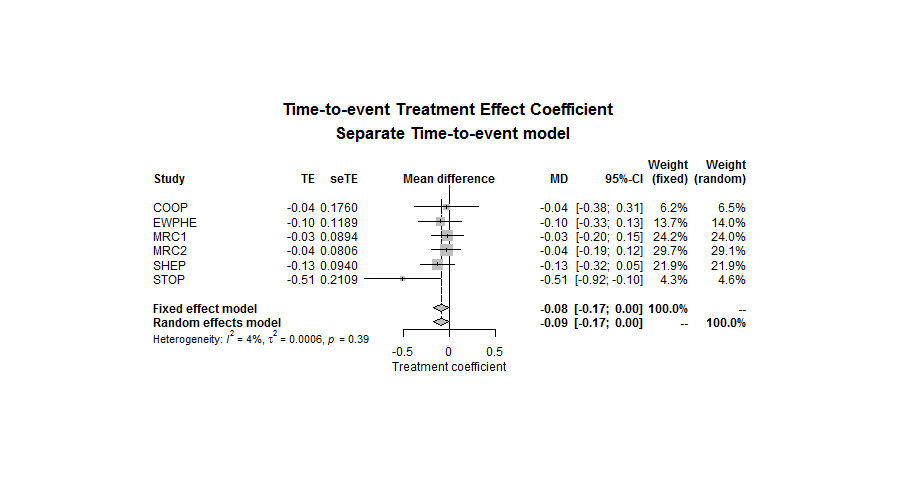


Supplemental Figure 6: Forest plot for time-to-event treatment effect covariate from standalone / separate time-to-event model for SBP and time to death

### Joint time-to-event treatment effect


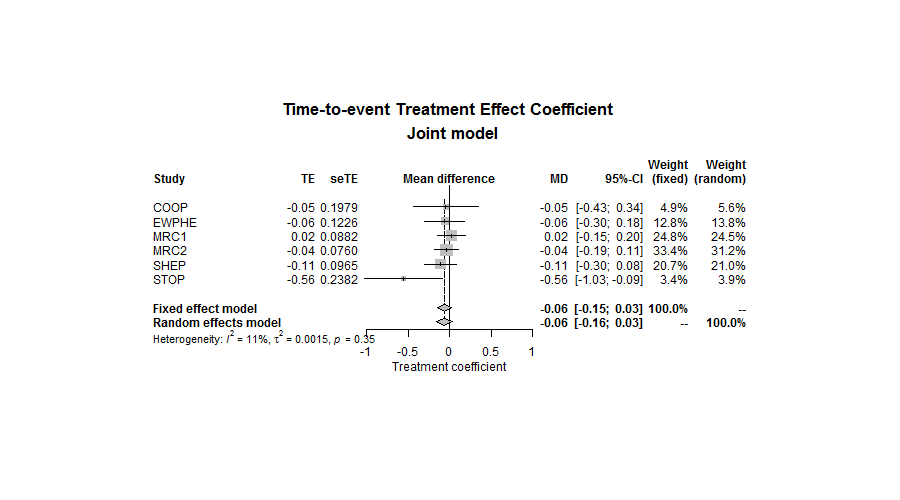


Supplemental Figure 7: Forest plot for time-to-event treatment effect covariate from joint model for SBP and time to death

### Association parameters


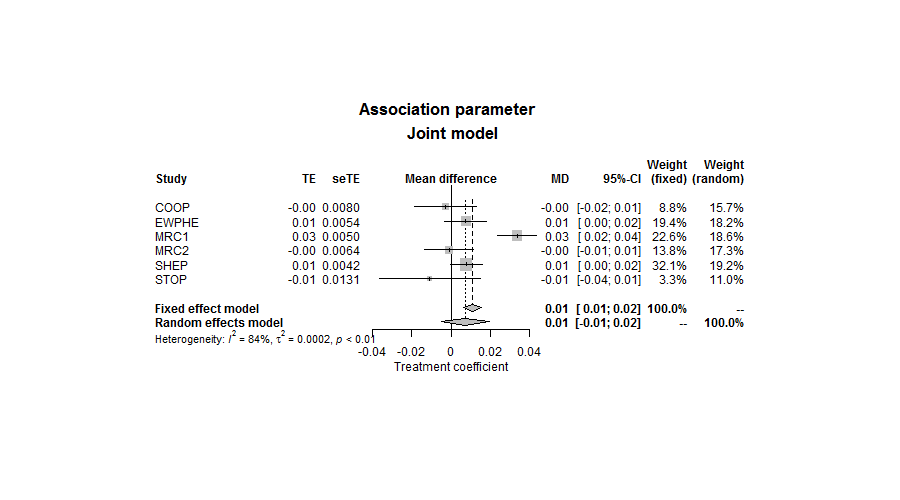


Supplemental Figure 8: Forest plot for association parameter from joint model for SBP and time to death

## SBP and time to MI

### Separate longitudinal treatment effect


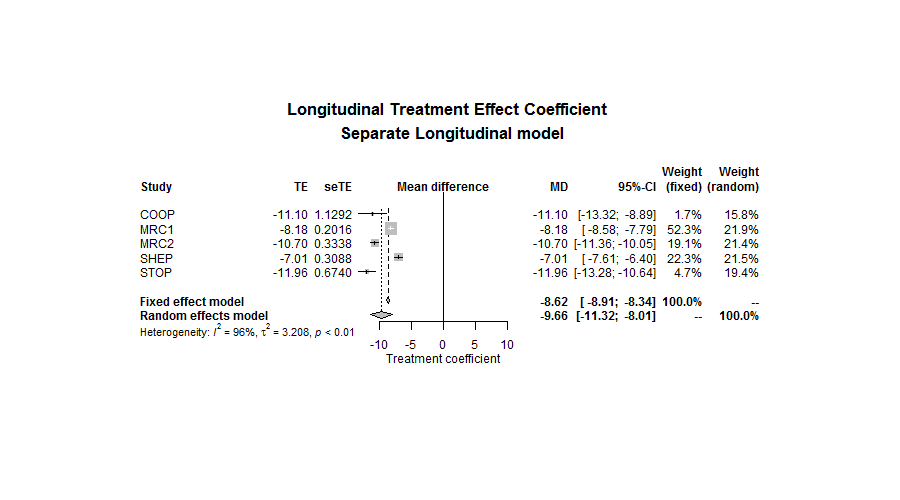


Supplemental Figure 9: Forest plot for longitudinal treatment effect covariate from standalone / separate longitudinal model for SBP and time to myocardial infarction (MI)

### Joint longitudinal treatment effect


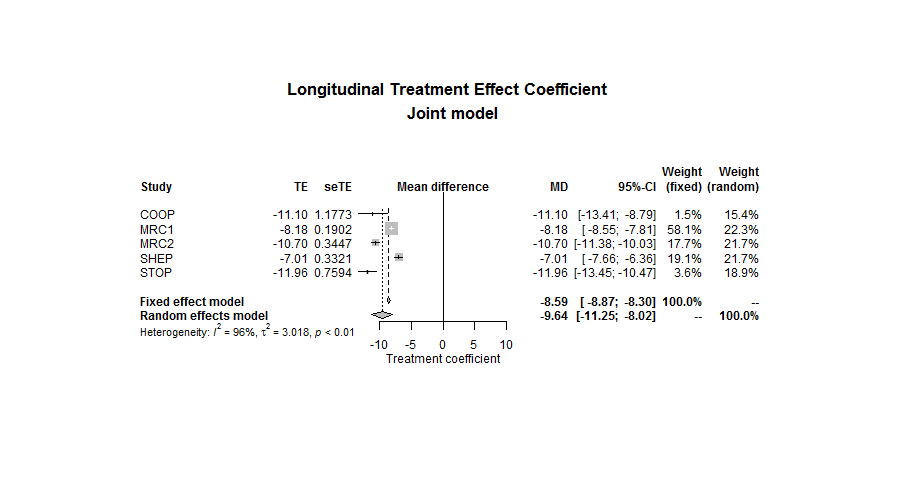


Supplemental Figure 10: Forest plot for longitudinal treatment effect covariate from joint model for SBP and time to myocardial infarction (MI)

### Separate time-to-event treatment effect


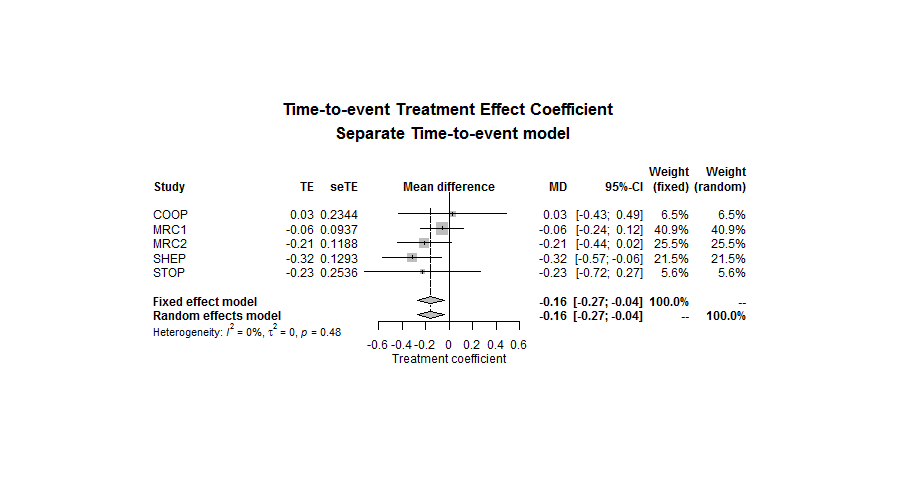


Supplemental Figure 11: Forest plot for time-to-event treatment effect covariate from standalone / separate time-to-event model for SBP and time to myocardial infarction (MI)

### Joint time-to-event treatment effect


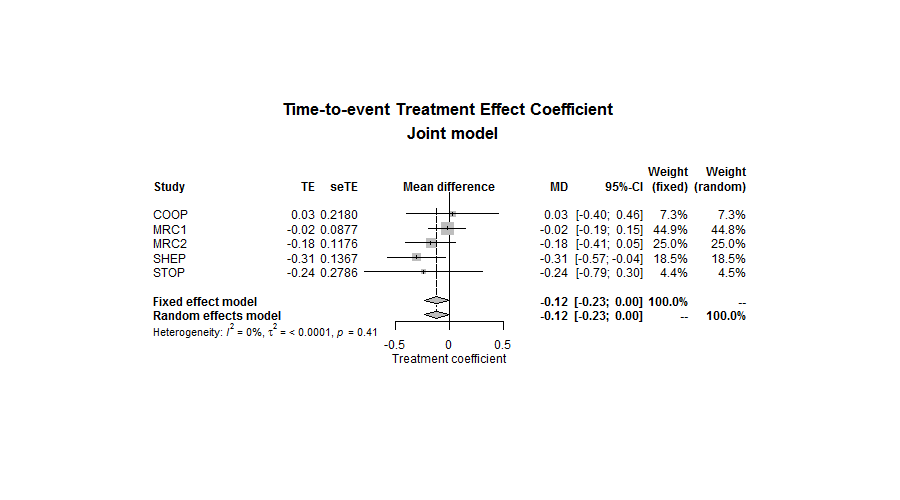


Supplemental Figure 12: Forest plot for time-to-event treatment effect covariate from joint model for SBP and time to myocardial infarction (MI)

### Association parameters


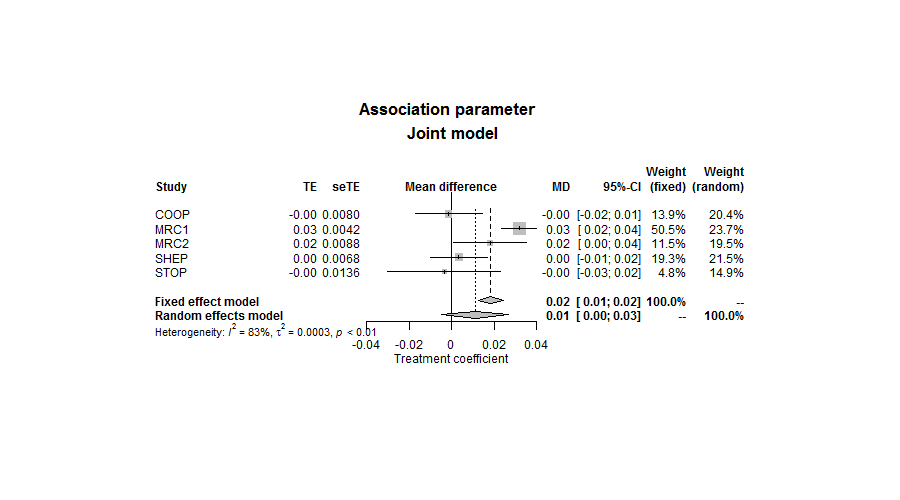


Supplemental Figure 13: Forest plot for association parameter from joint model for SBP and time to myocardial infarction (MI)

## SBP and time to ST

### Separate longitudinal treatment effect


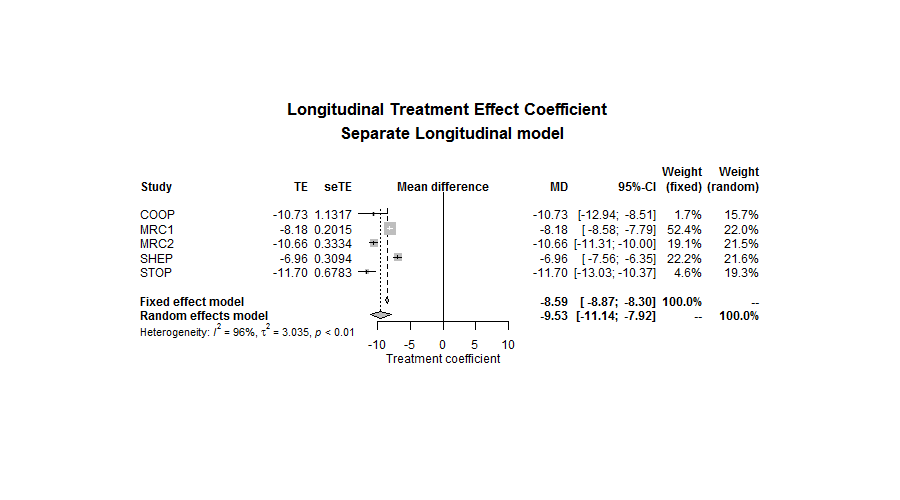


Supplemental Figure 14: Forest plot for longitudinal treatment effect covariate from standalone / separate longitudinal model for SBP and time to stroke

### Joint longitudinal treatment effect


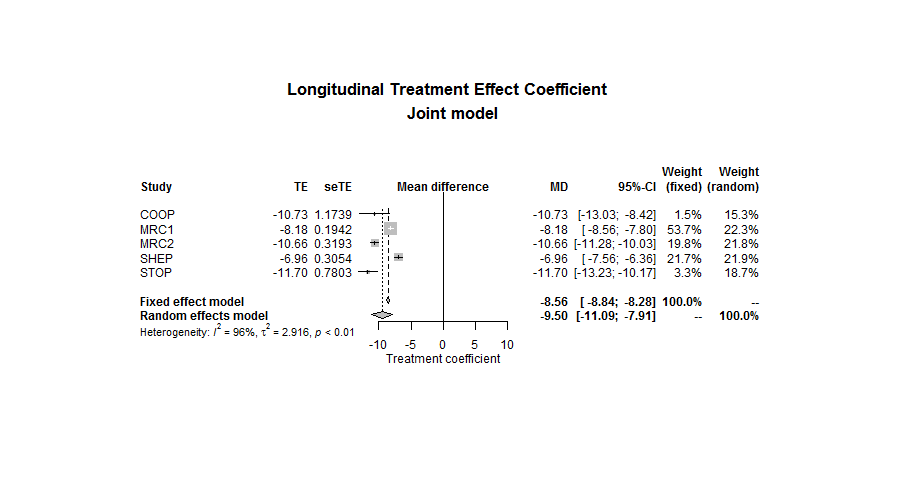


Supplemental Figure 15: Forest plot for longitudinal treatment effect covariate from joint model for SBP and time to stroke

### Separate time-to-event treatment effect


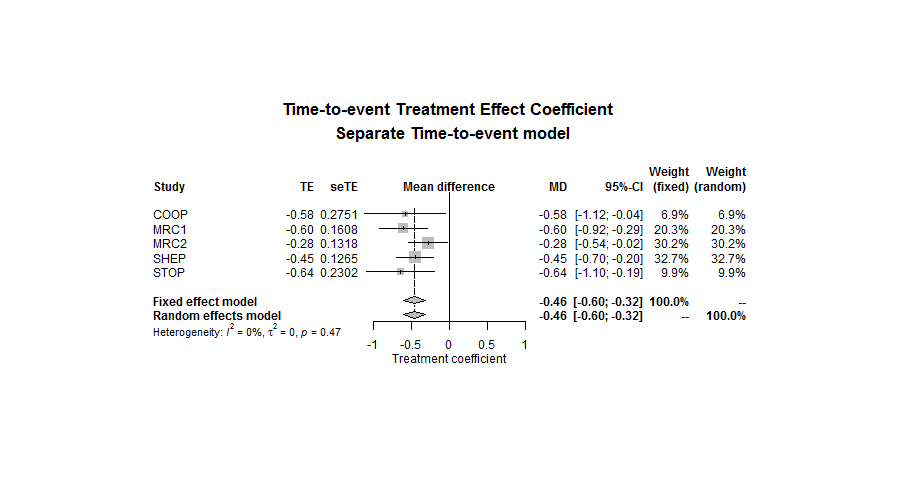


Supplemental Figure 16: Forest plot for time-to-event treatment effect covariate from standalone / separate time-to-event model for SBP and time to stroke

### Joint time-to-event treatment effect


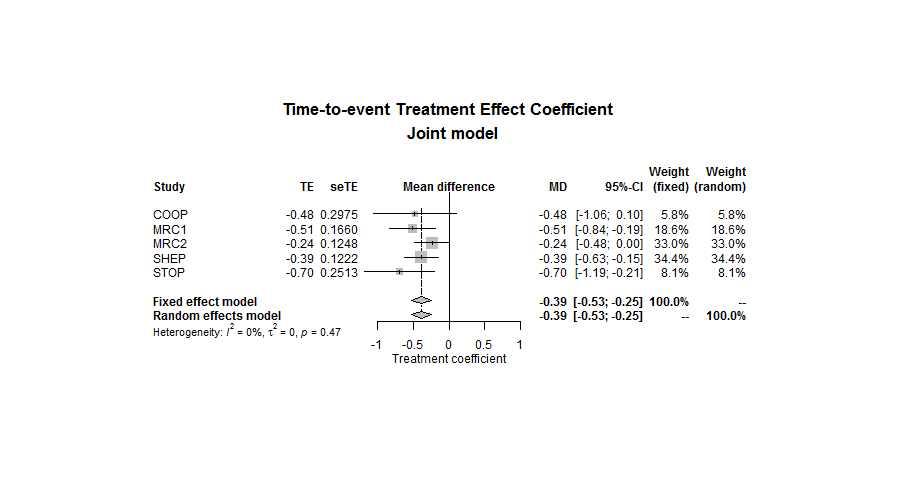


Supplemental Figure 17: Forest plot for time-to-event treatment effect covariate from joint model for SBP and time to stroke

### Association parameters


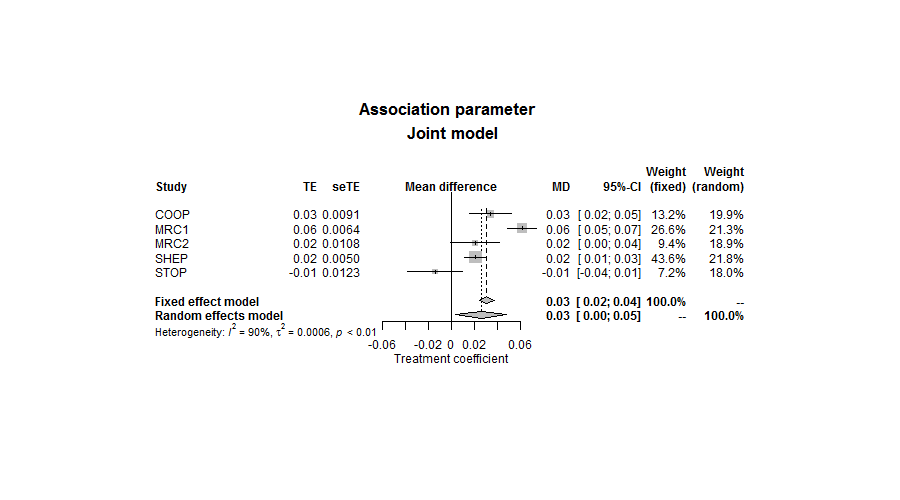


Supplemental Figure 18: Forest plot for association parameter from joint model for SBP and time to stroke

# Event rates in simulated data

|  | Association | Measures | Overall | Study 1 | Study 2 | Study 3 | Study 4 | Study 5 |
| --- | --- | --- | --- | --- | --- | --- | --- | --- |
| Low Event Rate Data | 0 | Mean (sd) | 24 (0.8) | 24 (1.9) | 24 (1.9) | 24.1 (1.9) | 24 (1.8) | 24 (1.9) |
|  |  | Median (LQ,UQ) | 24 (23.4,24.6) | 24 (22.8,25.2) | 24 (22.8,25.2) | 24.2 (22.9,25.6) | 24 (22.8,25.2) | 24 (22.8,25.4) |
|  | 0.25 | Mean (sd) | 23.9 (0.8) | 23.9 (1.8) | 23.9 (1.9) | 24 (1.9) | 23.9 (1.9) | 23.9 (1.9) |
|  |  | Median (LQ,UQ) | 23.9 (23.4,24.5) | 23.8 (22.8,25.2) | 24 (22.6,25.2) | 24 (22.6,25.2) | 23.8 (22.6,25.2) | 24 (22.8,25.2) |
|  | 0.5 | Mean (sd) | 24 (0.9) | 24 (1.9) | 23.9 (1.9) | 23.9 (1.9) | 24 (1.9) | 24 (2) |
|  |  | Median (LQ,UQ) | 24 (23.4,24.5) | 24 (22.8,25.4) | 23.8 (22.6,25.2) | 24 (22.8,25.2) | 23.8 (22.8,25.2) | 24 (22.6,25.4) |
|  | 0.75 | Mean (sd) | 24.3 (0.9) | 24.3 (2) | 24.3 (1.8) | 24.4 (1.9) | 24.4 (2) | 24.2 (1.9) |
|  |  | Median (LQ,UQ) | 24.3 (23.7,25) | 24.4 (23,25.6) | 24.2 (23.2,25.6) | 24.3 (23,25.8) | 24.4 (23,25.8) | 24.2 (23,25.4) |
|  | 1 | Mean (sd) | 24 (0.9) | 24 (1.9) | 23.9 (1.9) | 23.9 (1.9) | 24 (1.9) | 24 (2) |
|  |  | Median (LQ,UQ) | 24 (23.4,24.5) | 24 (22.8,25.4) | 23.8 (22.6,25.2) | 24 (22.8,25.2) | 23.8 (22.8,25.2) | 24 (22.6,25.4) |
| High Event rate data | 0 | Mean (sd) | 87 (0.7) | 87 (1.6) | 87 (1.5) | 87 (1.5) | 86.9 (1.6) | 87 (1.5) |
|  |  | Median (LQ,UQ) | 87 (86.5,87.4) | 87 (86,88) | 87 (86,88) | 87 (86,88.2) | 87 (85.8,88) | 87 (86,88) |
|  | 0.25 | Mean (sd) | 86.6 (0.7) | 86.6 (1.5) | 86.6 (1.5) | 86.6 (1.5) | 86.6 (1.5) | 86.5 (1.5) |
|  |  | Median (LQ,UQ) | 86.6 (86.1,87) | 86.6 (85.6,87.6) | 86.6 (85.6,87.6) | 86.6 (85.6,87.6) | 86.6 (85.6,87.6) | 86.6 (85.6,87.6) |
|  | 0.5 | Mean (sd) | 84.3 (0.7) | 84.3 (1.7) | 84.4 (1.7) | 84.3 (1.5) | 84.3 (1.6) | 84.3 (1.7) |
|  |  | Median (LQ,UQ) | 84.3 (83.8,84.8) | 84.4 (83.2,85.4) | 84.4 (83.2,85.4) | 84.2 (83.2,85.4) | 84.4 (83.2,85.4) | 84.4 (83.2,85.6) |
|  | 0.75 | Mean (sd) | 79.6 (0.8) | 79.6 (1.8) | 79.6 (1.8) | 79.6 (1.8) | 79.6 (1.8) | 79.7 (1.8) |
|  |  | Median (LQ,UQ) | 79.7 (79.1,80.2) | 79.6 (78.4,80.8) | 79.6 (78.4,81) | 79.6 (78.4,80.8) | 79.6 (78.4,80.8) | 79.8 (78.6,81) |
|  | 1 | Mean (sd) | 75 (0.9) | 75 (1.9) | 75 (2) | 74.9 (2.1) | 75.1 (1.9) | 74.9 (1.9) |
|  |  | Median (LQ,UQ) | 75 (74.4,75.5) | 75 (73.6,76.2) | 75 (73.6,76.2) | 74.8 (73.6,76.4) | 75.2 (73.8,76.4) | 74.8 (73.6,76.2) |

Supplemental Table 4: Mean and median event rates for homogeneous scenarios

|  | Association | Measures | Overall | Study 1 | Study 2 | Study 3 | Study 4 | Study 5 |
| --- | --- | --- | --- | --- | --- | --- | --- | --- |
| Low Event Rate Data | 0 | Mean (sd) | 24.1 (1) | 24.1 (2.1) | 24.1 (2.2) | 24.2 (2.1) | 24.2 (2.1) | 24.1 (2.1) |
|  |  | Median (LQ, UQ) | 24.1 (23.5,24.8) | 24.1 (22.6,25.6) | 24.2 (22.6,25.6) | 24.2 (22.8,25.6) | 24.2 (22.8,25.6) | 24 (22.6,25.6) |
|  | 0.25 | Mean (sd) | 24 (0.9) | 23.9 (2.2) | 24 (2.2) | 24 (2.1) | 24 (2.2) | 24.1 (2.1) |
|  |  | Median (LQ, UQ) | 24 (23.4,24.6) | 23.8 (22.4,25.4) | 24 (22.6,25.6) | 24 (22.6,25.4) | 24 (22.6,25.4) | 24 (22.6,25.6) |
|  | 0.5 | Mean (sd) | 24.1 (0.9) | 24.1 (2.2) | 24.1 (2) | 24.2 (2.1) | 24.1 (2.2) | 24.1 (2.1) |
|  |  | Median (LQ, UQ) | 24.1 (23.5,24.8) | 24 (22.6,25.6) | 24 (22.6,25.6) | 24.2 (22.8,25.6) | 24 (22.6,25.6) | 24 (22.8,25.6) |
|  | 0.75 | Mean (sd) | 24.4 (0.9) | 24.5 (2.2) | 24.4 (2.1) | 24.4 (2) | 24.4 (2.1) | 24.3 (2.2) |
|  |  | Median (LQ, UQ) | 24.4 (23.8,25) | 24.6 (23.1,26) | 24.4 (23,25.8) | 24.4 (23,25.8) | 24.4 (23,25.8) | 24.2 (22.8,25.8) |
|  | 1 | Mean (sd) | 25 (0.9) | 25 (2.1) | 25.1 (2.1) | 25.1 (2.2) | 25.2 (2.1) | 24.9 (2.1) |
|  |  | Median (LQ, UQ) | 25 (24.4,25.7) | 25 (23.8,26.4) | 25 (23.6,26.4) | 25 (23.6,26.6) | 25 (23.8,26.4) | 24.9 (23.6,26.2) |
| High Event rate data | 0 | Mean (sd) | 87 (0.7) | 87 (1.5) | 87 (1.5) | 87 (1.5) | 87 (1.4) | 87 (1.5) |
|  |  | Median (LQ, UQ) | 87 (86.6,87.5) | 87 (86,88) | 87 (86,88) | 87 (86,88.2) | 87 (86.2,88) | 87 (86,88) |
|  | 0.25 | Mean (sd) | 86.6 (0.7) | 86.6 (1.5) | 86.6 (1.5) | 86.6 (1.5) | 86.6 (1.5) | 86.6 (1.5) |
|  |  | Median (LQ, UQ) | 86.6 (86.1,87) | 86.6 (85.6,87.6) | 86.6 (85.6,87.6) | 86.6 (85.6,87.6) | 86.6 (85.6,87.8) | 86.6 (85.6,87.6) |
|  | 0.5 | Mean (sd) | 84.3 (0.7) | 84.3 (1.6) | 84.4 (1.6) | 84.5 (1.6) | 84.3 (1.6) | 84.3 (1.5) |
|  |  | Median (LQ, UQ) | 84.4 (83.9,84.8) | 84.2 (83.4,85.4) | 84.4 (83.4,85.4) | 84.4 (83.4,85.6) | 84.2 (83.2,85.6) | 84.4 (83.2,85.4) |
|  | 0.75 | Mean (sd) | 79.7 (0.8) | 79.7 (1.8) | 79.6 (1.8) | 79.6 (1.8) | 79.6 (1.8) | 79.8 (1.7) |
|  |  | Median (LQ, UQ) | 79.7 (79.2,80.2) | 79.6 (78.6,80.8) | 79.6 (78.4,80.8) | 79.6 (78.4,80.8) | 79.6 (78.4,80.8) | 79.8 (78.6,81) |
|  | 1 | Mean (sd) | 75 (0.9) | 74.9 (2) | 75 (1.9) | 75 (1.9) | 74.9 (2) | 75.1 (1.9) |
|  |  | Median (LQ, UQ) | 74.9 (74.4,75.6) | 75 (73.6,76.2) | 75 (73.6,76.2) | 75 (73.8,76.4) | 74.8 (73.4,76.2) | 75.2 (73.8,76.4) |

Supplemental Table 5: Mean and median event rates for heterogeneous treatment scenarios

# Two stage simulations results tables

Across all simulations and scenarios, there were no failed fits for any separate model or joineR model.

## Homogenous low event rate

### Joint model

| Model | | Scenario | | | | |
| --- | --- | --- | --- | --- | --- | --- |
|  |  | 9 : $\boldsymbol{\alpha}=\boldsymbol{0}$ | 10: $\boldsymbol{\alpha}=\boldsymbol{0}.\boldsymbol{25}$ | 1: $\boldsymbol{\alpha}=\boldsymbol{0}.\boldsymbol{5}$ | 11: $\boldsymbol{\alpha}=\boldsymbol{0}.\boldsymbol{75}$ | 2: $\boldsymbol{\alpha}=\boldsymbol{1}$ |
| Joint longitudinal and time-to-event model (JoineR, proportional association, shared random effects, unspecified baseline hazard) | | | | | | |
| Longitudinal treatment effect coefficient | Fixed | 2.00 (0.04) {94.9} | 2.00 (0.04) {94.3} | 2.01 (0.04) {92.6} | 2.02 (0.04) {92.9} | 2.02 (0.04) {89.4} |
|  | Bias Fixed | 0.00 (0.04) | 0.00 (0.04) | 0.01 (0.04) | 0.02 (0.04) | 0.02 (0.04) |
|  | Random | 2.00 (0.04) {96.5} | 2.00 (0.04) {95.7} | 2.01 (0.04) {94.4} | 2.02 (0.04) {94.1} | 2.02 (0.04) {91.8} |
|  | Bias Random | 0.00 (0.04) | 0.00 (0.04) | 0.01 (0.04) | 0.02 (0.04) | 0.02 (0.04) |
|  | $\tau^{2}$ | 0.02 (0.03) | 0.02 (0.03) | 0.02 (0.03) | 0.02 (0.03) | 0.03 (0.04) |
| Time-to-event treatment effect coefficient (log{HR}) | Fixed | 2.92 (0.15) {92.1} | 2.94 (0.14) {93.6} | 2.96 (0.13) {94.1} | 2.95 (0.13) {92.7} | 2.93 (0.14) {89.2} |
|  | Bias Fixed | -0.08 (0.15) | -0.06 (0.14) | -0.04 (0.13) | -0.05 (0.13) | -0.07 (0.14) |
|  | Random | 2.93 (0.14) {94.2} | 2.95 (0.14) {95.2} | 2.97 (0.13) {95.7} | 2.96 (0.13) {94.8} | 2.93 (0.14) {91.3} |
|  | Bias Random | -0.07 (0.14) | -0.05 (0.14) | -0.03 (0.13) | -0.04 (0.13) | -0.07 (0.14) |
|  | $\tau^{2}$ | 0.05 (0.11) | 0.08 (0.12) | 0.09 (0.13) | 0.09 (0.13) | 0.09 (0.13) |
| Association estimate | Fixed | 0.00 (0.01) {93.8} | 0.25 (0.02) {95.6} | 0.50 (0.02) {93.2} | 0.74 (0.03) {93.1} | 0.98 (0.03) {88.9} |
|  | Bias Fixed | 0.00 (0.01) | 0.00 (0.02) | 0.00 (0.02) | -0.01 (0.03) | -0.02 (0.03) |
|  | Random | 0.00 (0.01) {95.9} | 0.25 (0.02) {97.1} | 0.50 (0.02) {95.9} | 0.74 (0.03) {94.7} | 0.98 (0.03) {92.6} |
|  | Bias Random | 0.00 (0.01) | 0.00 (0.02) | 0.00 (0.02) | -0.01 (0.03) | -0.02 (0.03) |
|  | $\tau^{2}$ | 0.01 (0.01) | 0.01 (0.02) | 0.01 (0.02) | 0.02 (0.02) | 0.02 (0.03) |

Supplemental Table 6: Two stage simulation results homogenous low event rate data joint model (joineR)

### Separate models

| Model | | Scenario | | | | |
| --- | --- | --- | --- | --- | --- | --- |
|  |  | 9 : $\boldsymbol{\alpha}=\boldsymbol{0}$ | 10: $\boldsymbol{\alpha}=\boldsymbol{0}.\boldsymbol{25}$ | 1: $\boldsymbol{\alpha}=\boldsymbol{0}.\boldsymbol{5}$ | 11: $\boldsymbol{\alpha}=\boldsymbol{0}.\boldsymbol{75}$ | 2: $\boldsymbol{\alpha}=\boldsymbol{1}$ |
| Longitudinal model | | | | | | |
| Longitudinal treatment effect coefficient | Fixed | 2.00 (0.03) {95.1} | 2.00 (0.04) {94.4} | 2.01 (0.04) {92.6} | 2.02 (0.04) {92.1} | 2.03 (0.04) {87.4} |
|  | Bias Fixed | 0.00 (0.03) | 0.00 (0.04) | 0.01 (0.04) | 0.02 (0.04) | 0.03 (0.04) |
|  | Random | 2.00 (0.03) {96.5} | 2.00 (0.04) {95.7} | 2.01 (0.04) {94.4} | 2.02 (0.04) {93.2} | 2.03 (0.04) {90.2} |
|  | Bias Random | 0.00 (0.03) | 0.00 (0.04) | 0.01 (0.04) | 0.02 (0.04) | 0.03 (0.04) |
|  | $\tau^{2}$ | 0.02 (0.03) | 0.02 (0.03) | 0.02 (0.03) | 0.02 (0.03) | 0.03 (0.04) |
| Time-to-event model | | | | | | |
| Time-to-event treatment effect coefficient (log{HR}) | Fixed | 2.98 (0.15) {93.3} | 2.37 (0.13) {0.2} | 1.79 (0.11) {0.0} | 1.49 (0.09) {0.0} | 1.30 (0.09) {0.0} |
|  | Bias Fixed | -0.02 (0.15) | -0.63 (0.13) | -1.21 (0.11) | -1.51 (0.09) | -1.70 (0.09) |
|  | Random | 3.00 (0.15) {95.5} | 2.39 (0.13) {2.7} | 1.80 (0.11) {0.0} | 1.50 (0.09) {0.0} | 1.30 (0.09) {0.0} |
|  | Bias Random | 0.00 (0.15) | -0.61 (0.13) | -1.20 (0.11) | -1.50 (0.09) | -1.70 (0.09) |
|  | $\tau^{2}$ | 0.10 (0.14) | 0.12 (0.14) | 0.08 (0.10) | 0.06 (0.09) | 0.06 (0.08) |

Supplemental Table 7: Two stage simulation results homogenous low event rate data separate models

## Homogenous high event rate

### Joint model

| Model | | Scenario | | | | |
| --- | --- | --- | --- | --- | --- | --- |
|  |  | **12:** $\boldsymbol{\alpha=0}$ | **13:** $\boldsymbol{\alpha=0.25}$ | **3:** $\boldsymbol{\alpha=0.5}$ | **14:** $\boldsymbol{\alpha=0.75}$ | **4:** $\boldsymbol{\alpha=1}$ |
| Joint longitudinal and time-to-event model (JoineR, proportional association, shared random effects, unspecified baseline hazard) | | | | | | |
| Longitudinal treatment effect coefficient | Fixed | 2.00 (0.03) {94.8} | 2.01 (0.03) {95.5} | 2.01 (0.04) {92.2} | 2.02 (0.04) {89.2} | 2.03 (0.03) {89.1} |
|  | Bias Fixed | 0.00 (0.03) | 0.01 (0.03) | 0.01 (0.04) | 0.02 (0.04) | 0.03 (0.03) |
|  | Random | 2.00 (0.03) {96.6} | 2.01 (0.03) {96.0} | 2.01 (0.04) {94.6} | 2.02 (0.04) {91.9} | 2.03 (0.03) {91.7} |
|  | Bias Random | 0.00 (0.03) | 0.01 (0.03) | 0.01 (0.04) | 0.02 (0.04) | 0.03 (0.03) |
|  | $\tau^{2}$ | 0.02 (0.03) | 0.02 (0.03) | 0.02 (0.03) | 0.02 (0.03) | 0.02 (0.03) |
| Time-to-event treatment effect coefficient (log{HR}) | Fixed | 2.99 (0.08) {93.5} | 2.99 (0.08) {94.3} | 2.98 (0.07) {93.7} | 2.98 (0.08) {92.7} | 2.97 (0.08) {93.2} |
|  | Bias Fixed | -0.01 (0.08) | -0.01 (0.08) | -0.02 (0.07) | -0.02 (0.08) | -0.03 (0.08) |
|  | Random | 3.00 (0.08) {95.1} | 2.99 (0.07) {96.0} | 2.98 (0.07) {95.4} | 2.98 (0.08) {94.4} | 2.98 (0.08) {95.8} |
|  | Bias Random | 0.00 (0.08) | -0.01 (0.07) | -0.02 (0.07) | -0.02 (0.08) | -0.02 (0.08) |
|  | $\tau^{2}$ | 0.05 (0.08) | 0.05 (0.07) | 0.05 (0.07) | 0.05 (0.08) | 0.06 (0.08) |
| Association estimate | Fixed | 0.00 (0.01) {96.0} | 0.25 (0.01) {94.4} | 0.50 (0.01) {91.1} | 0.74 (0.01) {89.7} | 0.98 (0.02) {84.4} |
|  | Bias Fixed | 0.00 (0.01) | 0.00 (0.01) | 0.00 (0.01) | -0.01 (0.01) | -0.02 (0.02) |
|  | Random | 0.00 (0.01) {96.9} | 0.25 (0.01) {96.2} | 0.50 (0.01) {93.8} | 0.74 (0.01) {92.3} | 0.98 (0.02) {88.5} |
|  | Bias Random | 0.00 (0.01) | 0.00 (0.01) | 0.00 (0.01) | -0.01 (0.01) | -0.02 (0.02) |
|  | $\tau^{2}$ | 0.004 (0.01) | 0.004 (0.01) | 0.01 (0.01) | 0.01 (0.01) | 0.01 (0.02) |

Supplemental Table 8: Two stage simulation results homogenous high event rate data joint model (joineR)

Separate models

| Model | | Scenario | | | | |
| --- | --- | --- | --- | --- | --- | --- |
|  |  | **12:** $\boldsymbol{\alpha=0}$ | **13:** $\boldsymbol{\alpha=0.25}$ | **3:** $\boldsymbol{\alpha=0.5}$ | **14:** $\boldsymbol{\alpha=0.75}$ | **4:** $\boldsymbol{\alpha=1}$ |
| Longitudinal model | | | | | | |
| Longitudinal treatment effect coefficient | Fixed | 2.00 (0.03) {95.0} | 2.01 (0.03) {95.1} | 2.02 (0.04) {90.4} | 2.03 (0.04) {86.2} | 2.04 (0.03) {82.0} |
|  | Bias Fixed | 0.00 (0.03) | 0.01 (0.03) | 0.02 (0.04) | 0.03 (0.04) | 0.04 (0.03) |
|  | Random | 2.00 (0.03) {96.4} | 2.01 (0.03) {95.7} | 2.02 (0.04) {93.0} | 2.03 (0.04) {88.9} | 2.04 (0.03) {85.8} |
|  | Bias Random | 0.00 (0.03) | 0.01 (0.03) | 0.02 (0.04) | 0.03 (0.04) | 0.04 (0.03) |
|  | $\tau^{2}$ | 0.02 (0.03) | 0.02 (0.03) | 0.02 (0.03) | 0.02 (0.03) | 0.02 (0.03) |
| Time-to-event model | | | | | | |
| Time-to-event treatment effect coefficient (log{HR}) | Fixed | 3.00 (0.08) {94.3} | 2.01 (0.08) {0.0} | 1.18 (0.06) {0.0} | 0.85 (0.05) {0.0} | 0.69 (0.05) {0.0} |
|  | Bias Fixed | 0.00 (0.08) | -0.99 (0.08) | -1.82 (0.06) | -2.15 (0.05) | -2.31 (0.05) |
|  | Random | 3.01 (0.08) {95.3} | 2.02 (0.08) {0.0} | 1.19 (0.06) {0.0} | 0.85 (0.05) {0.0} | 0.69 (0.05) {0.0} |
|  | Bias Random | 0.01 (0.08) | -0.98 (0.08) | -1.81 (0.06) | -2.15 (0.05) | -2.31 (0.05) |
|  | $\tau^{2}$ | 0.05 (0.08) | 0.10 (0.09) | 0.06 (0.06) | 0.04 (0.05) | 0.04 (0.05) |

Supplemental Table 9: Two stage simulation results homogenous high event rate data separate models

## Heterogeneous treatment low event rate

### Joint model

| Model | | Scenario | | | | |
| --- | --- | --- | --- | --- | --- | --- |
|  |  | 9 : $\boldsymbol{\alpha}=\boldsymbol{0}$ | 10: $\boldsymbol{\alpha}=\boldsymbol{0}.\boldsymbol{25}$ | 1: $\boldsymbol{\alpha}=\boldsymbol{0}.\boldsymbol{5}$ | 11: $\boldsymbol{\alpha}=\boldsymbol{0}.\boldsymbol{75}$ | 2: $\boldsymbol{\alpha}=\boldsymbol{1}$ |
| Joint longitudinal and time-to-event model (JoineR, proportional association, shared random effects, unspecified baseline hazard) | | | | | | |
| Longitudinal treatment effect coefficient | Fixed | 2.01 (0.23) {26.6} | 2.00 (0.22) {26.0} | 2.01 (0.23) {22.8} | 2.01 (0.22) {24.9} | 2.02 (0.24) {23.4} |
|  | Bias Fixed | 0.01 (0.23) | 0.00 (0.22) | 0.01 (0.23) | 0.01 (0.22) | 0.02 (0.24) |
|  | Random | 2.01 (0.23) {87.3} | 2.00 (0.22) {89.2} | 2.01 (0.23) {86.6} | 2.01 (0.22) {89.3} | 2.02 (0.23) {86.6} |
|  | Bias Random | 0.01 (0.23) | 0.00 (0.22) | 0.01 (0.23) | 0.01 (0.22) | 0.02 (0.23) |
|  | $\tau^{2}$ | 0.47 (0.18) | 0.46 (0.18) | 0.47 (0.17) | 0.47 (0.18) | 0.47 (0.18) |
| Time-to-event treatment effect coefficient (log{HR}) | Fixed | 2.76 (0.27) {58.5} | 2.84 (0.26) {63.6} | 2.89 (0.25) {66.8} | 2.86 (0.26) {62.5} | 2.84 (0.27) {56.9} |
|  | Bias Fixed | -0.24 (0.27) | -0.16 (0.26) | -0.11 (0.25) | -0.14 (0.26) | -0.16 (0.27) |
|  | Random | 2.84 (0.24) {82.2} | 2.92 (0.25) {86.8} | 2.97 (0.25) {90.6} | 2.94 (0.25) {88.0} | 2.92 (0.27) {86.2} |
|  | Bias Random | -0.16 (0.24) | -0.08 (0.25) | -0.03 (0.25) | -0.06 (0.25) | -0.08 (0.27) |
|  | $\tau^{2}$ | 0.28 (0.25) | 0.34 (0.25) | 0.41 (0.25) | 0.43 (0.25) | 0.44 (0.25) |
| Association estimate | Fixed | 0.00 (0.01) {95.8} | 0.25 (0.02) {95.1} | 0.49 (0.02) {95.1} | 0.74 (0.03) {93.9} | 0.98 (0.03) {87.9} |
|  | Bias Fixed | 0.00 (0.01) | 0.00 (0.02) | -0.01 (0.02) | -0.01 (0.03) | -0.02 (0.03) |
|  | Random | 0.00 (0.01) {96.7} | 0.25 (0.02) {96.5} | 0.50 (0.02) {96.2} | 0.74 (0.03) {95.2} | 0.98 (0.02 ){91.0} |
|  | Bias Random | 0.00 (0.01) | 0.00 (0.02) | 0.00 (0.02) | -0.01 (0.03) | -0.02 (0.03) |
|  | $\tau^{2}$ | 0.01 (0.01) | 0.01 (0.02) | 0.01 (0.02) | 0.01 (0.02) | 0.02 (0.03) |

Supplemental Table 10: Two stage simulation results heterogeneous treatment low event rate data joint model (joineR)

Separate models

| Model | | Scenario | | | | |
| --- | --- | --- | --- | --- | --- | --- |
|  |  | 9 : $\boldsymbol{\alpha}=\boldsymbol{0}$ | 10: $\boldsymbol{\alpha}=\boldsymbol{0}.\boldsymbol{25}$ | 1: $\boldsymbol{\alpha}=\boldsymbol{0}.\boldsymbol{5}$ | 11: $\boldsymbol{\alpha}=\boldsymbol{0}.\boldsymbol{75}$ | 2: $\boldsymbol{\alpha}=\boldsymbol{1}$ |
| Longitudinal model | | | | | | |
| Longitudinal treatment effect coefficient | Fixed | 2.01 (0.23) {26.1} | 2.00 (0.22) {25.6} | 2.01 (0.23) {22.1} | 2.01 (0.22) {24.7} | 2.03 (0.23) {24.4} |
|  | Bias Fixed | 0.01 (0.23) | 0.00 (0.22) | 0.01 (0.23) | 0.01 (0.22) | 0.03 (0.23) |
|  | Random | 2.01 (0.23) {87.0} | 2.00 (0.22) {89.2} | 2.01 (0.23) {86.8} | 2.01 (0.22) {89.3} | 2.03 (0.23) {86.6} |
|  | Bias Random | 0.01 (0.23) | 0.00 (0.22) | 0.01 (0.23) | 0.01 (0.22) | 0.03 (0.23) |
|  | $\tau^{2}$ | 0.47 (0.18) | 0.46 (0.18) | 0.47 (0.17) | 0.47 (0.18) | 0.47 (0.18) |
| Time-to-event model | | | | | | |
| Time-to-event treatment effect coefficient (log{HR}) | Fixed | 2.86 (0.27) {64.3} | 2.32 (0.23) {4.2) | 1.78 (0.16) {0.0} | 1.47 (0.14) {0.0} | 1.28 (0.12) {0.0} |
|  | Bias Fixed | -0.14 (0.27) | -0.68 (0.23) | -1.22 (0.16) | -1.53 (0.14) | -1.72 (0.12) |
|  | Random | 2.98 (0.26) {88.7} | 2.40 (0.23) {25.8} | 1.81 (0.17) {0.0} | 1.49 (0.14) {0.0} | 1.29 (0.12) {0.0} |
|  | Bias Random | -0.02 (0.26) | -0.60 (0.23) | -1.19 (0.17) | -1.51 (0.14) | -1.71 (0.12) |
|  | $\tau^{2}$ | 0.42 (0.26) | 0.36 (0.21) | 0.26 (0.17) | 0.20 (0.15) | 0.16 (0.13) |

Supplemental Table 11: Two stage simulation results heterogeneous treatment low event rate data separate models

## Heterogeneous treatment high event rate

### Joint model

| Model | | Scenario | | | | |
| --- | --- | --- | --- | --- | --- | --- |
|  |  | **12:** $\boldsymbol{\alpha=0}$ | **13:** $\boldsymbol{\alpha=0.25}$ | **3:** $\boldsymbol{\alpha=0.5}$ | **14:** $\boldsymbol{\alpha=0.75}$ | **4:** $\boldsymbol{\alpha=1}$ |
| Joint longitudinal and time-to-event model (JoineR, proportional association, shared random effects, unspecified baseline hazard) | | | | | | |
| Longitudinal treatment effect coefficient | Fixed | 1.99 (0.23) {20.6} | 2.02 (0.23) {23.5} | 2.02 (0.23) {22.0} | 2.01 (0.23) {20.3} | 2.02 (0.23) {21.7} |
|  | Bias Fixed | -0.01 (0.23) | 0.02 (0.23) | 0.02 (0.23) | 0.01 (0.23) | 0.02 (0.23) |
|  | Random | 2.00 (0.23) {88.2} | 2.02 (0.23) {88.0} | 2.02 (0.23) {85.9} | 2.01 (0.23) {88.1} | 2.02 (0.23) {87.2} |
|  | Bias Random | 0.00 (0.23) | 0.02 (0.23) | 0.02 (0.23) | 0.01 (0.23) | 0.02 (0.23) |
|  | $\tau^{2}$ | 0.47 (0.17) | 0.47 (0.18) | 0.47 (0.18) | 0.47 (0.17) | 0.47 (0.18) |
| Time-to-event treatment effect coefficient (log{HR}) | Fixed | 2.85 (0.24) {41.1} | 2.89 (0.25) {40.0} | 2.89 (0.25) {40.7} | 2.90 (0.25) {42.6} | 2.90 (0.25) {43.8} |
|  | Bias Fixed | -0.15 (0.24) | -0.11 (0.25) | -0.11 (0.25) | -0.10 (0.25) | -0.10 (0.25) |
|  | Random | 2.98 (0.23) {88.4} | 2.98 (0.24) {88.0} | 2.98 (0.24) {86.3} | 2.97 (0.24) {87.2} | 2.97 {0.25) {86.2} |
|  | Bias Random | -0.02 (0.23) | -0.02 (0.24) | -0.02 (0.024) | -0.03 (0.24) | -0.03 (0.25) |
|  | $\tau^{2}$ | 0.45 (0.20) | 0.44 (0.20) | 0.46 (0.20) | 0.45 (0.20) | 0.45 (0.21) |
| Association estimate | Fixed | 0.00 (0.01) {93.9} | 0.25 (0.01) {95.4} | 0.50 (0.01) {91.8} | 0.74 (0.01) {87.9} | 0.98 (0.02) {83.6} |
|  | Bias Fixed | 0.00 (0.01) | 0.00 (0.01) | 0.00 (0.01) | -0.01 (0.01) | -0.02 (0.02) |
|  | Random | 0.00 (0.01) {96.0} | 0.25 (0.01) {97.0} | 0.50 (0.01) {94.1} | 0.74 (0.01) {90.6} | 0.98 (0.02) {87.9} |
|  | Bias Random | 0.00 (0.01) | 0.00 (0.01) | 0.00 (0.01) | -0.01 (0.01) | -0.02 (0.02) |
|  | $\tau^{2}$ | 0.003 (0.01) | 0.005 (0.01) | 0.01 (0.01) | 0.01 (0.01) | 0.01 (0.02) |

Supplemental Table 12: Two stage simulation results heterogeneous treatment high event rate data joint model (joineR)

Separate models

| Model | | Scenario | | | | |
| --- | --- | --- | --- | --- | --- | --- |
|  |  | **12:** $\boldsymbol{\alpha=0}$ | **13:** $\boldsymbol{\alpha=0.25}$ | **3:** $\boldsymbol{\alpha=0.5}$ | **14:** $\boldsymbol{\alpha=0.75}$ | **4:** $\boldsymbol{\alpha=1}$ |
| Longitudinal model | | | | | | |
| Longitudinal treatment effect coefficient | Fixed | 2.00 (0.23) {22.1} | 2.02 (0.23) {23.5} | 2.03 (0.23) {21.8} | 2.02 (0.23) {20.1} | 2.03 (0.23) {22.6} |
|  | Bias Fixed | 0.00 (0.23) | 0.02 (0.23) | 0.03 (0.23) | 0.02 (0.23) | 0.03 (0.23) |
|  | Random | 2.00 (0.23) {88.6} | 2.02 (0.23) {88.3} | 2.03 (0.23) {85.7} | 2.02 (0.23) {88.3) | 2.03 (0.23) {87.1} |
|  | Bias Random | 0.00 (0.23) | 0.02 (0.23) | 0.03 (0.23) | 0.02 (0.23) | 0.03 (0.23) |
|  | $\tau^{2}$ | 0.47 (0.17) | 0.47 (0.18) | 0.47 (0.18) | 0.47 (0.17) | 0.47 (0.18) |
| Time-to-event model | | | | | | |
| Time-to-event treatment effect coefficient (log{HR}) | Fixed | 2.86 (0.24) {40.1} | 1.97 (0.19) {0.0} | 1.18 (0.11) {0.0} | 0.85 (0.08) {0.0} | 0.69 (0.07) {0.0} |
|  | Bias Fixed | -0.14 (0.24) | -1.03 (0.19) | -1.82 (0.11) | -2.15 (0.08) | -2.31 (0.07) |
|  | Random | 2.99 (0.23) {88.2} | 2.03 (0.20) {0.9} | 1.19 (0.11) {0.0} | 0.85 (0.08) {0.0} | 0.69 (0.07) {0.0} |
|  | Bias Random | -0.01 (0.23) | -0.97 (0.20) | -1.81 (0.11) | -2.15 (0.08) | -2.31 (0.07) |
|  | $\tau^{2}$ | 0.45 (0.20) | 0.36 (0.15) | 0.19 (0.10) | 0.12 (0.08) | 0.09 (0.08) |

Supplemental Table 13: Two stage simulation results heterogeneous treatment high event rate data separate models
